# Mechanistic profiling and optimized production of Altenusin, a fungal carboxy-biphenyl scaffold for tyrosinase inhibition

**DOI:** 10.1039/d5ra09904h

**Published:** 2026-02-19

**Authors:** Nicolas Reyes Castillo, Marius Spohn, Celine M. Zumkeller, Michael Marner, Yang Liu, Maria A. Patras, Christian Kersten, Francesca Magari, Arnold Grünweller, Till F. Schäberle

**Affiliations:** a Natural Product Research, Institute for Insect Biotechnology, Justus-Liebig-University Giessen 35392 Gießen Germany till.f.schaeberle@agrar.uni-giessen.de; b Fraunhofer Institute for Molecular Biology and Applied Ecology, Branch of Bioresources Ohlebergsweg 12 35392 Gießen Germany marius.spohn@ime.fraunhofer.de; c Institute of Pharmaceutical and Biomedical Sciences, Johannes Gutenberg-University Mainz Staudinger Weg 5 55128 Mainz Germany; d Institute for Quantitative and Computational Bioscience, Johannes Gutenberg-University Mainz BioZentrum l, Hanns-Dieter-Hüsch Weg 15 55128 Mainz Germany; e Institute of Pharmaceutical Chemistry, Philipps-University Marburg Marbacher Weg 6 35032 Marburg Germany; f German Center for Infection Research (DZIF), Partner Site Gießen-Marburg-Langen Ohlebergsweg 12 35392 Gießen Germany

## Abstract

Tyrosinase is a binuclear copper oxidase central to melanogenesis and food browning and is a major target for depigmenting and anti-browning agents. Here we evaluate Altenusin, a fungal carboxy-biphenyl polyketide, as a tyrosinase-inhibitor scaffold by combining structure-based screening, enhanced fermentation and mechanistic enzymology. Docking against the mushroom tyrosinase *Agaricus bisporus* PPO_3_ (AbPPO_3_) highlighted Altenusin as a presumed dicopper-site binder, and genome mining of the producer strain revealed a polyketide synthase gene cluster consistent with its biosynthesis. Fermentation optimization and bioreactor transfer increased Altenusin titers up to 0.254 ± 0.022 g L^−1^. *In vitro*, Altenusin inhibited in a substrate-dependent manner, with IC_50_ values of 0.381 ± 0.002 mM (l-tyrosine) and 0.162 ± 0.023 mM (l-DOPA); kinetic analysis indicated competitive monophenolase inhibition and mixed-type diphenolase inhibition. Altenusin also showed strong radical-scavenging and copper-reducing activity, moderate Cu^2+^ chelation and a narrow cytotoxicity window in HepG2 cells (48 h, CC_50_: 0.093 mM). Overall, these data define Altenusin as a biotechnologically tractable starting point for fungal carboxy-biphenyl inhibitor discovery.

## Abstract 2

An integrated upstream/downstream-to-mechanism workflow supports altenusin as a scalable, assay-robust tyrosinase-inhibitor scaffold for anti-melanosis strategies.

## Graphical abstract

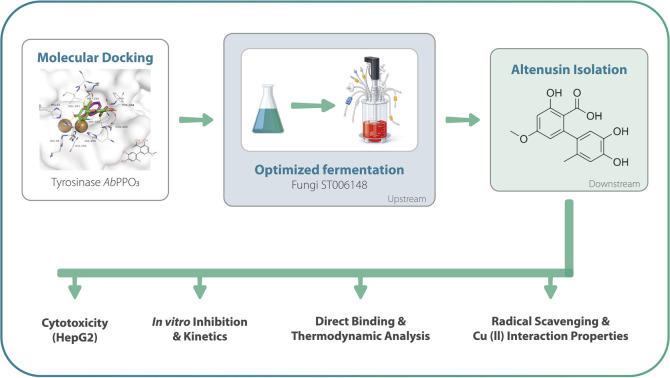

## Introduction

Tyrosinase (EC 1.14.18.1) is a copper-containing oxidase widely distributed in multicellular organisms, where it catalyzes two essential reactions in melanogenesis: the *ortho*-hydroxylation of monophenols, such as l-tyrosine into *o*-diphenols like l-DOPA, and their subsequent oxidation into reactive *o*-quinones.^1,2^ These *o*-quinones polymerize into melanin that acts as a natural photoprotector and camouflaging mechanism against ultraviolet radiation and other environmental hazards.^3^ However, excessive activity of this enzyme contributes to cutaneous hyperpigmentation disorders, such as melasma^4^ and drives enzymatic browning of foods,^5,6^ leading to undesirable discoloration and substantial post-harvest economic losses.^7^

Because of this central role, tyrosinase is a major target for cosmetic, dermatological and food-preservation applications, and numerous natural products, including phenolic acids, flavonoids, stilbenes and coumarins have been reported as inhibitors.^5,8^ Yet, many of these scaffolds suffer from intrinsic liabilities that limit efficacy or complicate their incorporation into stable formulations, such as UV-induced *trans*-cis isomerization (*e.g.*, *p*-coumaric and ferulic acids^9,10^), auto-oxidation (chalcones and hydroxystilbenes, such as resveratrol^11–14^), poor aqueous solubility (*e.g.*, glabridin^15^) or formation of insoluble metal complexes (*e.g.*, ellagic acid^16,17^). Even kojic acid, the current benchmark fungal metabolite used in cosmetic formulations, is associated with skin sensitization and photoinstability in aqueous systems.^18^ These issues underscore the need for new tyrosinase inhibitor scaffolds that combine robust copper binding with chemical stability and formulation-friendly physicochemical properties.

Rational discovery of such improved scaffolds requires leveraging structural information on the tyrosinase active site. Structural studies of mushroom tyrosinase (*Agaricus bisporus*, PDB: 2Y9X^2^) have revealed a binuclear copper center composed of two copper ions, denoted as CuA and CuB, in which each copper ion is coordinated with three conserved histidine residues – His61, His85 and His94 for CuA and His259, His263 and His296 for CuB – defining a type-III copper oxidase with both mono- and diphenolase activities.^1,2^ Inhibitors, such as tropolone, chelate both copper ions and establish hydrogen bonds and π–π contacts with active-site histidines (His259 and His263).^19,20^ Together, these binding modes illustrate how small, rigid aromatic ligands can chelate and effectively block the dicopper center.^8,21^

The structural data mentioned above suggest that potent tyrosinase inhibitors should combine a chelating motif for the dicopper center with a rigid, planar aromatic core that can stack against active-site residues. Biphenyl scaffolds fulfill these criteria: they are rigid, largely planar and can host multiple *ortho*- and *para*-substituted phenolic groups.^22^ The plant-derived biphenyl fortuneanoside D, for example, inhibits mushroom tyrosinase with an IC_50_ of 0.07 mM.^23^ Furthermore, several synthetic series of biphenyls have been prepared and evaluated as tyrosinase inhibitors, with the most active analogues also achieving IC_50_ values in the mid-micromolar range.^24–27^ Notably, structurally related biphenyl polyketides are also characteristic metabolites from the genus *Alternaria* spp.^28^ However, at least to our knowledge, fungal biphenyl natural products have not been systematically investigated as tyrosinase inhibitors, yet. Fungal-derived natural products already provide relevant tyrosinase inhibitors: *e.g.*, kojic acid (IC_50_: 0.062–0.121 mM ^29,30^) from *Aspergillus* spp., terrein from *Aspergillus terreus*, isoflavones from *Aspergillus oryzae*, and diverse metabolites from *Penicillium* and *Trichoderma* species with low micromolar potency.^30^ These examples highlight fungi as an exceptional, yet underexploited, source of structurally diverse tyrosinase inhibitors and as attractive hosts for sustainable, scalable fermentative production.

Motivated by the convergence between biphenyl-based tyrosinase inhibitors and the occurrence of biphenyl polyketides in *Alternaria* spp., we performed a chemoinformatic scan on Sanofi's 2K NP library of pure microbially-derived compounds.^31^ This revealed the fungal biphenyl natural product Altenusin, a hydroxylated carboxy-biphenyl isolated from multiple *Alternaria* species,^32–35^ as potential tyrosinase binder. Given the structural hallmarks shared with known plant-derived and synthetic tyrosinase inhibitors, *e.g.*, including a phenolic biphenyl scaffold and multiple *ortho*-hydroxyl groups, we hypothesized that Altenusin could display tyrosinase inhibitory activity and assessed both, its biochemical profile and its fermentative production.

In this study, we used the fungal polyketide Altenusin as a prototypical carboxy-biphenyl scaffold to characterize the interaction of this chemical class with the dicopper active site of tyrosinase. Specifically, we defined its biochemical inhibition profile against mushroom tyrosinase; elucidated its binding mode by kinetic analysis, fluorescence spectroscopy and molecular docking; quantified its antioxidant and copper-chelating/reducing activities; and assessed its cytotoxicity in mammalian cells. In addition, we applied a Box–Behnken design and bioreactor scale-up to demonstrate that an alternariol-like polyketide gene cluster in strain ST006148 enables scalable, milligram-scale production of the compound. Together, these experiments define Altenusin as a structurally and biotechnologically tractable fungal carboxy-biphenyl tyrosinase inhibitor with a well-characterized biochemical profile and a preliminary cellular safety window.

## Materials and methods

### Molecular docking studies

Molecular docking was performed using SeeSAR-11.0.0 ^36,37^ and the *Agaricus bisporus* PPO_3_ – tropolone complex structure (PDB-ID: 2Y9X^2^, chain B). The docking setup was validated by re-docking of the crystallographic reference ligand tropolone (Fig. S13A, RMSD = 2.1 Å) and binder-decoy discrimination by receiver operating characteristics area under the curve (ROC AUC = 0.79, Fig. S13B) analysis. 191 reported PPO ligands with affinity below 1 µM (pChEMBL-value ≥6 corresponding to IC_50_, *K*_D_, *etc.* ≤1 µM) were derived from ChEMBL (https://www.ebi.ac.uk/chembl/, accessed November 27th, 2021 ^38^). Property-matched decoys were generated using the Database for useful decoys enhanced (DUD-E^39^). Molecular docking for Altenusin pose prediction was performed using the same setup.

### Genomic DNA extraction, sequencing and assembly

DNA was isolated from mycelial biomass of the production strain ST006148 (Altenusin-producing fungal isolate) using the E.Z.N.A.® HP Fungal DNA Kit (Omega BIO-TEL, D2485). Illumina sequencing generated 2.7 Gb of data; sequencing reads were quality-filtered to remove adapters and low-quality bases prior to genome assembly. BUSCO^40^ (version 5.7.1) was employed to assess genome completeness, selecting the lineage dataset of dothideomycetes_odb10 in mode euk_genome_min. FungiSMASH^41^ 6.1.1 was used for BGC prediction in default mode, including option --cb-knownclusters, --cc-mibig --cb-general for comparison with known BGCs. Corason^42^ (default mode) was used for alignment of Alternariol/Altenusin-like BGCs.

### Medium & pre-inoculum preparation

Medium 5189 was prepared by dissolving 20 g per L malt extract (ME), 10 g per L glucose (G), 2 g per L yeast extract (YE) (Carl Roth, Karlsruhe, Germany), and 0.5 g per L diammonium hydrogen phosphate (NH_4_)_2_HPO_4_ (DAP) (Sigma-Aldrich, St. Louis, U.S.A.) in deionized water and adjusting the pH to 6.0. Medium was subsequently autoclaved at 121 °C for 20 minutes.

The fungal pre-inoculum was prepared by transferring a 1 cm^2^ agar plug containing mycelium into a 300 mL Erlenmeyer flask with 100 mL of 5189 medium. The flask was incubated at 25 °C in an orbital shaker at 180 rpm for 5 days. After incubation, the fungal culture was homogenized using a disperser for 30 s at 12 500 rpm (T-25 basic Ultra-Turrax) (IKA, Staufen, Germany) before being used for inoculation.

### Experimental design

The Design of Experiments (DoE) was carried out using Design-Expert v. 23.1.1 (Stat-Ease, Inc., Minneapolis, Minnesota, USA). Response Surface Methodology (RSM) based on Box–Behnken Design (BBD) was selected because it requires fewer runs than a full factorial design while still allowing estimation of the second-order interactions.^43^ It was therefore employed to assess the effect of the four components of medium 5189 on the production of Altenusin (mg L^−1^). For this purpose, the ranges for the four experimental factors were set as four-fold lower and four-fold higher than the nominal concentrations of each component in the normal medium (ME: 5–80 g L^−1^, DAP: 0.125–2 g L^−1^, G: 2.5–40 g L^−1^ and YE: 0.5–8 g L^−1^); to allow a broad exploratory window in the RSM. No prior tuning was performed; ranges were chosen to cover potential extremes of nutrient availability. Each factor was evaluated at three coded levels (−1 = low, 0 = center, +1 = high).

### Implementation and preliminary analysis

The BBD included 24 randomized runs corresponding to the factorial points (each combination evaluated once, with no additional replicates) and 5 center-point replicates (all factors at mid-level) to estimate the pure experimental error, for a total of 29 runs. Lack-of-fit was evaluated by ANOVA, and a *p*-value < 0.05 was considered statistically significant. Desirability was calculated using the Derringer–Suich methodology implemented in Design-Expert v.23.1.1., assigning equal weights to all factors and without modifying the software's default settings. The composite desirability index was obtained directly from Design-Expert's internal function, which combines the responses into a single metric ranging from 0 (not desirable) to 1 (fully desirable). To verify model assumptions, we inspected the diagnostic plots generated by the program; normal plot of residuals, to assess approximate normality and residuals *vs.* predicted values plot, to evaluate homoscedasticity and detect systematic patterns. No significant deviations from normality or heteroscedasticity were observed.

### Flask fermentation

Flask fermentations were carried out in 300 mL Erlenmeyer flasks containing 100 mL of 5189 medium or the optimized RSM-BBD medium (10% v/v pre-inoculum, 25 °C, 180 rpm) were sampled every 24 h up to 120 h, and at each time point cultures were harvested, lyophilized and extracted with 100% MeOH for HPLC-MS analysis for both the base 5189 and the optimized RSM-BBD medium.

### Batch fermentation setup

Batch fermentation was carried out in 1.7 L glass bioreactors (DASGIP SciVario, twin Spinner Vessels, Eppendorf, Hamburg, Germany) with a working volume of 1.5 L. The medium was gassed with filtered (0.22 µm) compressed air at a rate of 1 volume of air per volume of medium per minute (1 vvm), and the dissolved oxygen concentration was maintained at 30% throughout the process using an automatic control system. The temperature was controlled at 25 ± 1 °C, foam was restrained by adding Struktol J613A (0.015% v/v) (Schill + Seilacher, Hamburg, Germany), no pH adjustments were made during the process. Two media were tested in parallel: unmodified 5189 medium and the optimized RSM-BBD medium. The working volume was adjusted to 1.5 L with 5189 (or optimized) medium, and inoculated with approx. 10% (v/v) homogenized pre-inoculum (150 mL inoculum + 1.35 L medium). 10 mL samples for dry-weight determinations were taken at 0, 3, 16, 24, 27, 40, 48, 51, 64, 72, 75, 88, 96, 99, 112 and 120 h, which follow a repeating interval pattern of 3, 13 and 8 h (giving a 24 h cycle), to capture dry-weight and Altenusin production kinetics. Following the same time-course, parallel aliquots were withdrawn at each sampling point and processed for Altenusin quantification by UHPLC-MS, providing time-resolved production profiles for both the base 5189 and the optimized RSM-BBD medium.

### NMR and HPLC analysis

^1^H, ^13^C, and 2D NMR spectra were recorded at 25 °C in DMSO-*d*_6_ on Bruker Avance NEO 700 MHz (700 Neo) spectrometers. Chemical shifts were referenced to the solvent residual peaks, *δ*_H_ 2.50 (DMSO-*d*_6_) ppm for ^1^H, and *δ*_C_ 39.51 (DMSO-*d*_6_) ppm for ^13^C. HPLC was performed using a Shimadzu HPLC system (Shimadzu Deutschland GmbH, Duisburg, Germany) for analysis (EC 250/4.6 Nucleodur C18 Gravity SB, 5 µm; Macherey-Nagel, Düren, Germany), and for semi-preparative purification (VP 250/10 Nucleodur C18 Gravity-SB, 5 µm; Macherey-Nagel, Düren, Germany), and the following gradient was used (MeOH with 0.1% formic acid, H_2_O with 0.1% formic acid): 0 min (40% MeOH); 5 min (40% MeOH); 37 min (51% MeOH); 38 min (100% MeOH); 47 min (100% MeOH); 48 min (40% MeOH); 55 min (40% MeOH).

### Preparation of enzyme and Altenusin

Mushroom tyrosinase (Merck, Darmstadt, Germany) was dissolved in 0.1× PBS buffer (pH 6.8) (Thermo Fisher Scientific Inc., Waltham, U.S.A.) to a final activity of 600 U mL^−1^. Altenusin (Sigma-Aldrich, St. Louis, U.S.A.) was prepared at 3× the desired concentration in the same buffer, ensuring a constant final DMSO (Carl Roth, Karlsruhe, Germany) content below 5% (v/v) in all wells. 4-Hexylresorcinol (25 µM) (Sigma-Aldrich, St. Louis, U.S.A.) was included as positive control and buffer with 5% DMSO as vehicle (negative) control.

### Fluorescence quenching

Fluorescence quenching experiments were performed using a Cytation 5 multimode reader (BioTek Instruments, Winooski, VT, USA). Tyrosinase solutions (1000 U mL^−1^ in 50 mM phosphate buffer, pH 7.0) were titrated with increasing concentrations of Altenusin (0–640 µM). To each well, 75 µL of enzyme and 75 µL of inhibitor were mixed and incubated for 30 min at 25 °C before fluorescence emission spectra were recorded (*λ*_ex_: 280 nm; *λ*_em_: 300–700 nm). Steady-state quenching data were analyzed by plotting *F*_0_/*F versus* [Q] and fitting to the Stern–Volmer equation:
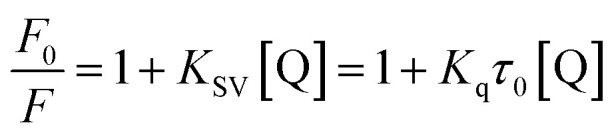
where, *F*_0_ and *F* are the fluorescence intensities of tyrosinase in the absence and presence of Altenusin, respectively. *K*_SV_ is the Stern–Volmer quenching constant, *K*_q_, the bimolecular quenching rate constant, *τ*_0_ the fluorescence lifetime and [Q] the quencher concentration. Subsequently, the apparent binding constant (*K*_a_) and the number of binding sites were determined by plotting and applying linear regression to the linearized double-logarithmic form of the static binding equation:
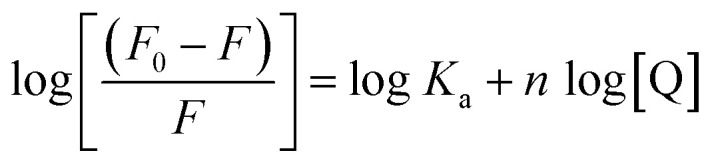


Thermodynamic parameters (Δ*H*° and Δ*S*°) were then extracted by applying the Van't Hoff equation:
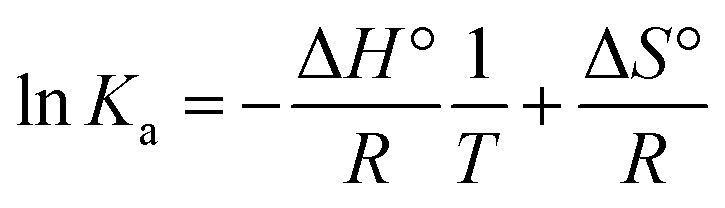


From which Δ*H*° and Δ*S*° were obtained by the slope and intercept of ln *K*_a_*versus T*^−1^. Finally, the standard free energy of Gibbs (binding energy) at each temperature was calculated according to:Δ*G*° = Δ*H*° − *T*Δ*S*°

### *In vitro* inhibition assay

The mono- and diphenolase tyrosinase assay was based on the method described by Nirmal & Benjakul,^44^ with slight modifications. All assays (total volume 150 µL) were carried out at 25 °C in a BMG LUMIstar OMEGA microplate reader (BMG Labtech, Ortenberg, Germany). To each well, 50 µL of Altenusin solution at varying concentrations was mixed with 50 µL of tyrosinase, and the mixture was pre-incubated for 10 min at 25 °C. The enzymatic reaction was started by adding 50 µL of substrate solution (4.5 mM l-DOPA for diphenolase activity or 4.5 mM l-tyrosine for monophenolase activity). Dopachrome formation was monitored at 475 nm at 14 s intervals over 45 min. One unit of polyphenol oxidase (PPO) activity was defined as the amount of enzyme producing an increase in *A*_475_ of 0.001 per minute per mL under standard conditions (pH 7.0, 25 °C). Initial reaction velocities (*ϑ*) were calculated from the linear portion of the absorbance-*versus*-time curves. Percent inhibition was calculated as:
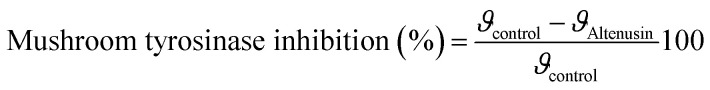
where, *ϑ*_control_ is the initial velocity of the control and *ϑ*_Altenusin_ was the initial velocity of Altenusin.

### Inhibition kinetics

Kinetic experiments were carried out in 150 µL reactions containing 50 µL mushroom tyrosinase (600 U mL^−1^ in 0.1× PBS), 50 µL Altenusin (final concentrations of 300, 350 and 400 µM for monophenolase and 100, 150 and 200 µM for diphenolase activity) and 50 µL substrate (l-DOPA or l-tyrosine (Sigma-Aldrich, St. Louis, U.S.A.) at 4.5, 4, 2, 1.33, 1, 0.8, 0.66 and 0.57 mM). After pre-incubating enzyme and inhibitor at 25 °C for 10 min, the reaction was initiated by adding the respective substrate and dopachrome formation was monitored at 475 nm every 14 s for 20 min on a BMG LUMIstar OMEGA microplate reader (BMG Labtech). Initial velocities (*ϑ*_0_) were obtained from the linear portion of each *A*_475_*vs.* time curve. The basic Lineweaver–Burk equation is:
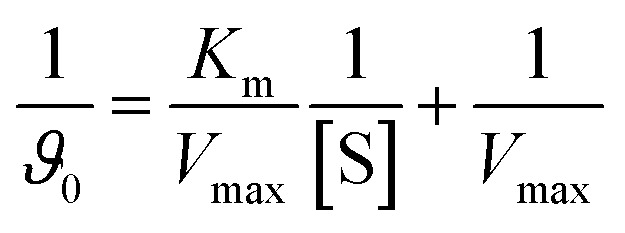


To establish the inhibition mechanism and obtain inhibition constants, initial rates were measured over a range of substrate concentrations at increasing inhibitor concentrations. Data from all inhibitor levels were globally fit by non-linear regression to Michaelis–Menten-based competitive, non-competitive and mixed-inhibition models. From the best-fitting model, we obtained apparent *K*_m_ and *V*_max_ values as well as inhibition constants *K*_i_ and 
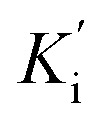
, corresponding to inhibitor binding to the free enzyme and to the enzyme–substrate complex, respectively. Lineweaver–Burk plots (*V*_0_^−1^*vs.* [S]^−1^) were generated from the same data and overlaid with curves calculated from the fitted parameters, and were used only as a qualitative check of the inhibition pattern.

### DPPH radical scavenging activity

1,1-Diphenyl-2-picrylhydrazyl (DPPH) method was conducted according to Zhang *et al.* (2020)^45^ and Latha *et al.* (2021)^46^ with slight modifications. Experiments were carried out in 200 µL reactions containing 100 µL DPPH Solution (0.3 mM) (Merck, Darmstadt, Germany) and 100 µL Altenusin (final concentrations of 1000, 800, 500, 400, 300, 200, 100, 50, 40, 30, 20, 10 and 5 µM). Trolox (0.4 mM) (6-hydroxy-2,5,7,8-tetramethylchroman-2-carboxylic acid) (Merck, Darmstadt, Germany) was used as positive control and 100% EtOH (Fisher, Hampton, U.S.A.) as the negative control. The absorbance was measured at 517 nm after incubation for 30 min in the dark at room temperature. Reactions were performed in triplicate and the inhibition of DPPH radical was calculated as follows:
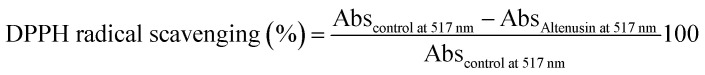
where, Abs_control_ is the absorbance of the negative control and Abs_Altenusin_ was the absorbance of Altenusin at different concentrations.

### ABTS radical scavenging activity

ABTS radical scavenging activity was determined by ABTS assay as described by Arnao *et al.* (2001)^47^ with slight modifications. The stock solutions included 7.4 mM ABTS solution (Selleckchem, Houston, U.S.A.) and 2.6 mM potassium persulfate solution (Sigma-Aldrich, St. Louis, U.S.A.). The working solution was prepared by mixing the two stock solutions in 1 : 1 relation and allowing them to react for 12–16 h at room temperature in the dark. The solution was then diluted by mixing 1 mL of ABTS solution with 50 mL of methanol in order to obtain an absorbance of 0.742 ± 0.02 units at 734 nm using a BMG LUMIstar OMEGA microplate reader (BMG Labtech, Ortenberg, Germany). Fresh ABTS solution was prepared for each assay. Samples (7.5 µL) were mixed with 142.5 µL of ABTS solution and the mixture was left at room temperature for 2 h in dark. The absorbance was then measured at 734 nm using the spectrophotometer. Trolox (0.4 mM) (6-hydroxy-2,5,7,8-tetramethylchroman-2-carboxylic acid) (Merck, Darmstadt, Germany) was used as positive control and 100% MeOH (Fisher, Hampton, U.S.A.) was used as negative control. Reactions were performed in triplicate and the inhibition of ABTS radical was calculated as follows:
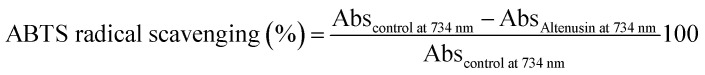
where, Abs_control_ is the absorbance of the negative control and Abs_Altenusin_ was the absorbance of Altenusin at different concentrations.

### Copper chelating capacity assay with pyrocatechol violet (PV)

Copper chelating method was carried out according to Latha *et al.* (2021)^46^ with slight modifications. Experiments were implemented in 200 µL reactions containing 50 µL CuSO_4_ solution (0.125 mM) (Sigma-Aldrich, St. Louis, U.S.A.), 50 µL Acetate buffer (50 mM, pH: 6.0) (Sigma-Aldrich, St. Louis, U.S.A.), 50 µL Altenusin (final concentrations of 5000, 4500, 4000, 3500, 3000, 2500, 1000, 800, 500, 400, 300, 200, 100, 50, 40, 30, 20, 10 and 5 µM) and PV (0.125 mM) (Carl Roth, Karlsruhe, Germany). EDTA (4 mM) (ethylenediaminetetraacetic acid) (Sigma-Aldrich, St. Louis, U.S.A.) was used as the positive control and acetate buffer as the negative control. The absorbance was measured at 632 nm after incubation at room temperature for 30 min. Reactions were performed in triplicate and copper chelating activity was calculated using the formula:
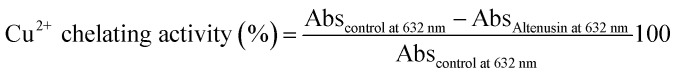
where, Abs_control_ is the absorbance of the negative control and Abs_sample_ was the absorbance of Altenusin at different concentrations.

### Copper(ii) reduction capacity assay with bathocuproine disulfonic acid (BCS)

Copper(ii) reduction method was carried out according to Campos *et al.* (2009)^48^ with slight modifications. Experiments were performed in 200 µL reactions containing 50 µL CuSO_4_ solution (0.5 mM), 50 µL Tris–HCl buffer (50 mM, pH: 7.4) (Carl Roth, Karlsruhe, Germany), 50 µL Altenusin (final concentrations of 3000, 2500, 1000, 800, 500, 400, 300, 200, 100, 80, 70, 60, 50, 40, 30, 20, 10, 5 and 1 µM) and BCS (0.2 mM) (Carl Roth, Karlsruhe, Germany). Ascorbic acid (0.4 mM) (Carl Roth, Karlsruhe, Germany) was used as the positive control and Tris–HCl buffer as the negative control. The absorbance was measured at 490 nm after incubation at room temperature for 10 min. Reactions were performed in triplicate and copper-reducing activity was calculated using the formula:
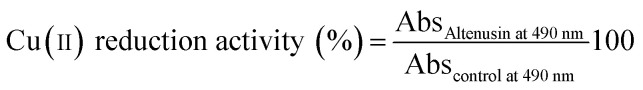
where, Abs_control_ is the absorbance of the positive control and Abs_Altenusin_ was the absorbance of Altenusin at different concentrations.

### Cytotoxicity assay

WST-1 assays (water-soluble tetrazolium assay) were performed to determine the toxicity of Altenusin in HepG2 cells (DSMZ no: ACC 180). HepG2 cells were cultivated in Iscove's Modified Dulbecco's Medium (Gibco, IMDM 1X). The CC_50_ value was determined by incubating 2 × 10^4^ HepG2 cells per well with 200 µL of compound (0, 10, 20, 30, 40, 50, 60, 70, 80, 90 and 100 µM) in a 96-well plate (Cellstar® 96-well Microplate, flat bottom clear polystyrene wells, Greiner Bio-One). After 48 h of incubation, WST-1 reagent (Art. No. 11644807001, Merck) was diluted 1 : 11 in PBS buffer (Gibco™ PBS 1X) and 110 µL was added to each well. After two hours of incubation at 37 °C and 5% CO_2_, cytotoxicity was measured at an absorbance wavelength of 450 nm in a Tecan microplate reader (Tecan Infinite M Plex) by using 600 nm as reference wavelength. To determine the CC_50_, WST-1 values were calculated in percentage and normalized with the respective DMSO control. CC_50_ values were calculated by non-linear regression analysis using GraphPad Prism 9.0 (GraphPad Software, San Diego, CA, USA). Standard error of the mean (SEM) was calculated for *n* ≥ 3.

### Statistical analysis

Unless otherwise stated, experiments were performed as at least three independent replicates. Data are reported as mean values with their corresponding dispersion (standard deviation, SD, or standard error of the mean, SEM, as indicated in the figure legends). Dose-response curves for IC_50_ and CC_50_ determinations, as well as enzyme inhibition assays, were fitted by non-linear regression to a four-parameter logistic model (4PL) using GraphPad Prism 9.0 (GraphPad Software, San Diego, CA, USA).

## Results and discussion

### Identification of Altenusin as a natural biphenyl scaffold for tyrosinase inhibition

A cheminformatic inspection of Sanofi's 2K NP library of microbial natural products^31^ ranked the fungal biphenyl Altenusin among the top candidate scaffolds for tyrosinase inhibition. To evaluate whether this *in silico* hit can productively engage the tyrosinase active site, we modeled Altenusin binding to the AbPPO_3_ structure (PDB: 2Y9X) at the dicopper catalytic center. We first benchmarked the docking protocol by re-docking the crystallographic reference inhibitor tropolone from the AbPPO_3_-ligand complex (PDB-ID: 2Y9X, re-docking RMSD = 2.1 Å, Fig. S13A) and binder-decoy discrimination (ROC-AUC: 0.79, Fig. S13B).

**Fig. 1:**
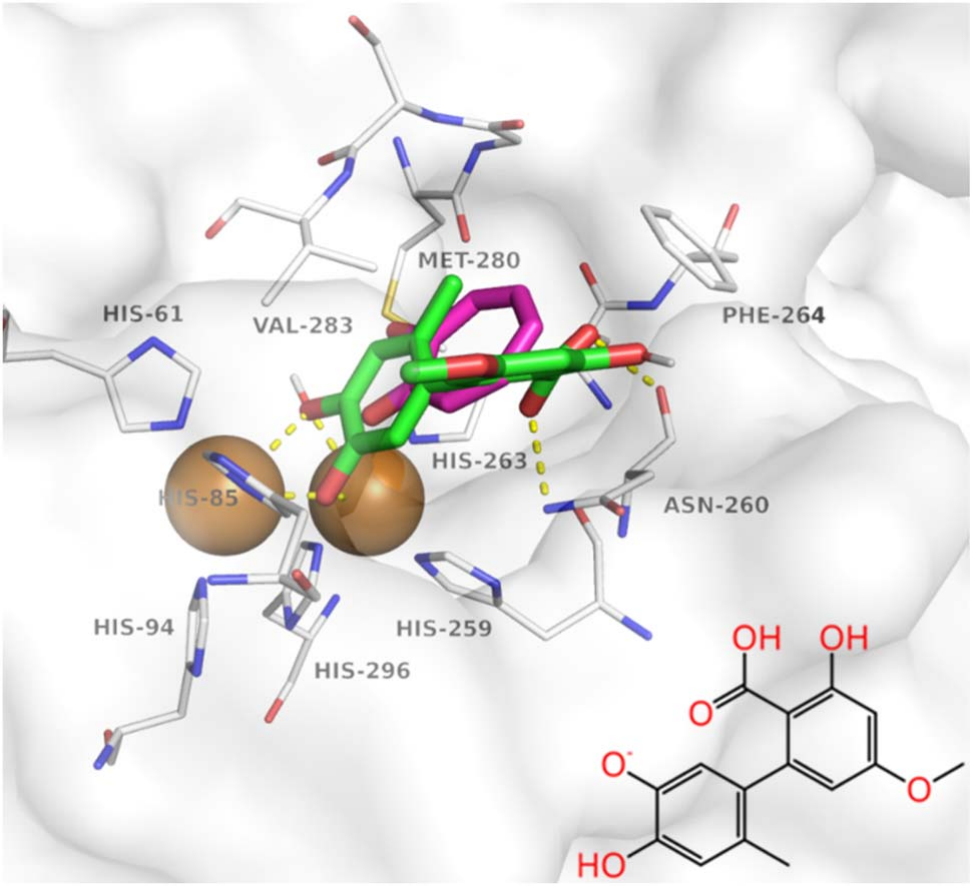
Docking predicted binding mode (green carbon atoms, HYDE-score: −37.5 kJ mol^−1^) and molecular structure of Altenusin in complex with AbPPO3 (white carbon atoms and transparent surface, PDB-ID: 2Y9X). Catalytic copper ions are shown as brown spheres, the crystallographic reference ligand is shown with magenta-colored carbon atoms for orientation, polar interactions are depicted as yellow dashed lines.

Based on the molecular docking-predicted binding mode (Fig. 1), Altenusin binds to AbPPO3 with its catechol substructure deprotonated for copper complexation. Additional hydrogen bonds are formed with the backbone and sidechain of Asn260 by the carboxylic acid which is found in its protonated state. Additional hydrophobic and stacking interactions are predicted with surrounding residues Val283, Met280, Phe264 and copper-binding His-residues 259, 263 and 85. These interactions and the predicted affinity (HYDE-score: −37.5 kJ mol^−1^) are similar to known tyrosinase inhibitors (Fig. S13 and S14). The dihedral angle between the two aromatic rings is close to optimal geometry with around 63°, compatible with a biphenyl catechol geometry. In line with design principles established for phenolic tyrosinase inhibitors bearing pre-organized catechol/cinnamic motifs,^49–51^ this pose supports Altenusin as a chemically plausible tyrosinase-inhibitor scaffold.

### Gene annotation and biosynthetic gene cluster homology

Motivated by these *in silico* data, we next examined the biosynthetic context of Altenusin production in its native producer ST006148 by sequencing its genomic DNA. The resulting assembly comprised 1344 contigs with a total size of 39.64 Mb, providing a basis for regulatory gene annotation and biosynthetic gene cluster (BGC) mining. This genome assembly shows an N50 of 172 kb and includes 96.1% complete BUSCOs, out of 3786 BUSCOs in the dothideomycetes_odb10 dataset (C: 96.1% [S: 96.1%, D: 0.0%], F: 0.4%, M: 3.5%, n: 3786, E: 2.3%). After confirming genome quality, fungiSMASH was used to predict 43 BGCs, comprising 20 PKS clusters, 12 NRPS clusters, 4 NRPS-like clusters, 5 NRPS-PKS hybrid clusters, 2 indole clusters, and 4 terpene clusters.

We focused on a BGC exhibiting high similarity to the MiBIG reference BGC0001284, encoding the synthesis of aromatic polyketides like alternariol, which is structurally related to Altenusin. Alternariol consists of two benzene rings fused to a γ-pyrone and is assembled by an iterative non-reducing type I polyketide synthase (NR-PKS). This NR-PKS follows the canonical architecture, starting with acetyl-CoA and extending with six malonyl-CoA units that are condensed *via* Claisen reactions. The PT domain then induces aldol-type cyclizations (between C-2/C-7 and C-8/C-13)^52^ to form the dibenzo-α-pyrone scaffold. The adjacent tailoring genes include *omtI* (*O*-methyltransferase), *moxI* (monooxygenase), *sdrI* (short-chain dehydrogenase), and *doxI* (extradiol dioxygenase). Together they are responsible for the biosynthesis of a plethora of related products, including alternariol-monomethyl ether, 4-hydroxy-alternariol monomethyl ether, Altenuene and Altenusin.^53,54^ The alternariol-like BGC found in the genome of strain ST006148 contains all the genes necessary for producing the secondary metabolites mentioned (Fig. S1). Consistent with the alternariol producer *Alternaria alternata*, strain ST006148 encodes an extra monooxygenase (SdrI) that is missing from the *Parastagonospora nodorum* BGC.^55^ It has been suggested that SdrI facilitates a reduction before lactone formation, creating an intermediate that eventually leads to Altenusin after methylation and hydroxylation at positions 5 and 5′, catalyzed by OmtI and MoxI, respectively. Indeed, it was shown before that deletion of *sdrI* or *moxI* prevents Altenusin production.^53^

### Altenusin production in shake flasks and stirred tank bioreactor

The alternariol-like polyketide BGC identified in strain ST006148 qualified this isolate for cultivation experiments, aimed at defining conditions that favor Altenusin biosynthesis over its derivatives. Guided by this genomic evidence, we conducted a focused OSMAC (one strain-many compounds) screen in shake flasks, systematically varying medium composition and cultivation parameters to map Altenusin output. Testing of medium compositions revealed a prioritized production regime that supported robust and reproducible Altenusin titers, which we subsequently adopted as the reference condition.

**Fig. 2:**
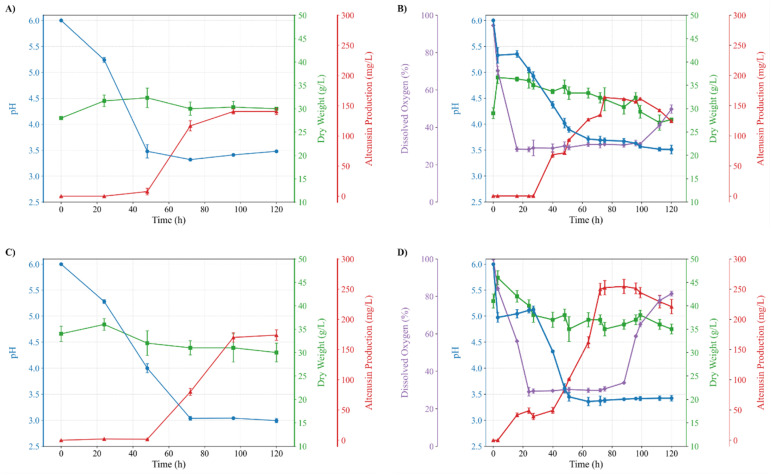
Kinetics of Altenusin production, biomass formation and cultivation parameters in shake flasks and a DO-controlled stirred-tank bioreactor. (A and B) Base production medium selected from the OSMAC screen: (A) shake flasks; time profiles of pH (left *y*-axis, blue), dry weight (DW; right *y*-axis, green) and Altenusin (right *y*-axis, red). (B) Stirred-tank bioreactor operated at a 30% DO setpoint; time profiles of pH (left *y*-axis, blue), dissolved oxygen (DO; left *y*-axis, purple), DW (right *y*-axis, green) and Altenusin (right *y*-axis, red). (C and D) Box–Behnken-optimized medium: (C) shake flasks and (D) stirred-tank bioreactor operated at a 30% DO setpoint, both showing higher and earlier Altenusin accumulation than the corresponding non-optimized cultures. Symbols and error bars represent mean ± SD (*n* = 3).

Under the selected production regime, we next quantified the temporal evolution of pH, biomass and Altenusin titers in shake flasks over the course of 120 h (Fig. 2A). The cultures acidified rapidly (from pH 6.0 to nearly 3.5 in approx. 40 h) and then remained stable. Biomass accumulated primarily during the first day, peaking at around 35 g L^−1^ at 50 h and stabilizing near 30 g L^−1^ thereafter. Altenusin appeared after growth deceleration and rose steadily to 0.140 ± 0.035 g L^−1^ by 112 h. This delayed post-growth accumulation is characteristic of non-growth-associated secondary metabolism in filamentous fungi, in which polyketide BGCs are preferentially expressed after the beginning of stationary phase and nutrient starvation.^56,57^

In the stirred-tank setup (Fig. 2B), pH likewise declined without control (from pH 6.0 to around 3.4 in approx. 70 h), with dissolved oxygen (DO) actively regulated to a 30% setpoint. DO dropped from air saturation to the setpoint within 24 to 40 h as biomass increased and was subsequently maintained near that level. Biomass reached around 33 to 35 g L^−1^ by 24 to 36 h and slowly settled near 30 g L^−1^. Notably, Altenusin accumulation initiated earlier (around 48 to 60 h) and achieved a higher, earlier maximum (0.160 to 0.170 g L^−1^ by 84 to 96 h) than in flasks, corresponding to a 15–20% higher peak titer in the bioreactor compared with flask cultures.

To further enhance Altenusin titers, we implemented a four-factor Box–Behnken response surface design in shake flasks, varying malt extract (ME), glucose (G), yeast extract (YE) and diammonium phosphate (DAP) (Table S1). The resulting quadratic model for Altenusin was highly significant (ANOVA: *F* = 14.07, *p* < 0.0001, Table S2) and showed good fit (*R*^2^ = 0.934, *R*_adj_^2^ = 0.867, *R*_pred_^2^ = 0.639, Table S3 and Fig. S2). YE exerted a positive linear effect on Altenusin (*p* = 0.0005), whereas DAP showed a negative linear effect (*p* < 0.0001). Instead, ME and G linear terms were not significant. All quadratic terms (ME^2^, G^2^, YE^2^, DAP^2^) were significant (*p* ≤ 0.0084), indicating interior optima and penalties upon over-supplementation (concave curvature). Lack-of-fit was borderline but not significant (*p* = 0.0705), which is acceptable given limited pure error.

Contour and surface plots (Fig. S3) at fixed ME = 20 g L^−1^ and G = 10 g L^−1^ visualized these trends: increasing YE to approx. 2 to 2.5 g L^−1^ boosts titers up, while DAP above 0.25 to 0.30 g L^−1^ decreases production – features captured by the significant C^2^ and D^2^ terms.^57^ The production pattern observed, with increased yields under yeast extract and repression under DAP, is consistent with the established role of C : N balance and nitrogen source in secondary metabolism of *Alternaria*.^58^ Acidic pH and organic nitrogen sources favor the biosynthesis of dibenzo-α-pyrones such as alternariol, while inorganic nitrogen, particularly ammonium or phosphate salts, strongly represses polyketide synthase activity. Excess nitrogen, whether organic or inorganic, diverts metabolism toward growth, as evidenced by the negative quadratic effects in the statistical model. Conversely, glucose appears an appropriate carbon source, sufficient to sustain growth without suppressing secondary metabolism.^59^ The single-response optimum (composite desirability D = 1.0) occurred at ME = 24.35 g L^−1^, G = 9.40 g L^−1^, YE = 2.055 g L^−1^ and DAP = 0.268 g L^−1^, with a model prediction of 0.1627 g per L Altenusin in flask scale setup. Based on this analysis, we selected an operating point by numerical desirability optimization in the sense of Derringer and Suich.^60^

Testing the optimized medium composition in shake-flask cultures (Fig. 2C) revealed similar pH and biomass trajectories as observed in the non-optimized medium, with biomass peaking around 36 g L^−1^ and pH declining from 6.0 to approx. 3.0 by 72 h. In contrast, Altenusin titers increased to 0.173 ± 0.088 g L^−1^ by 112 h, in good agreement with the model prediction (0.163 g L^−1^), while still displaying a clear post-growth, non-growth-associated production profile.

Relative to the initial fermentation reference regime, Altenusin titers increased from 0.140 to approx. 0.173 g L^−1^, corresponding to an approximate 30% gain in yield at essentially unchanged biomass. We next implemented the optimized medium in the stirred-tank bioreactor with the same setup as before (Fig. 2D). The DO profile again showed rapid oxygen consumption during the exponential phase, followed by a gradual rise as metabolic activity declined, whereas biomass reached 35–40 g L^−1^ and remained comparable to the non-optimized run. Under these conditions, Altenusin formation started earlier (by 18–24 h) and reached a maximum of 0.254 ± 0.022 g L^−1^ between 88 and 96 h. These gains are consistent with classic scale-up effects, whereby more uniform oxygen availability and narrower pellet-size distributions in stirred tanks reduce microenvironmental gradients that delay activation of polyketide pathways.^57,61^ Mechanistically, the kinetics agree with the linked regulation of fungal secondary metabolism, where physiological signals (C/N limitation, redox/DO, extracellular pH) reprogram transcription.^56,57^ Morphology likely contributed as well: stirred tanks typically narrow pellet-size distributions and improve effective O_2_ penetration across hyphal aggregates, boosting pathway activation and volumetric productivity.^60,62^ The transfer of the optimized medium formulation to the DO-controlled stirred-tank bioreactor revealed increased Altenusin peak titers of 0.254 g L^−1^, *i.e.*, an approximate 50 to 60% improvement relative to the initial bioreactor process executed with the unoptimized medium composition. This shows that medium composition and oxygen management act synergistically to enhance productivity at scale.

In submerged fermentation, the achieved titer of Altenusin (approx. 0.25 g L^−1^) lies within the upper range typically reported for specialized fungal polyketides obtained from non- or only modestly engineered strains after medium and process optimization, but remains well below titers achieved for bulk industrial metabolites.^57,63^ By comparison, kojic acid, a structurally simpler polyketide widely exploited as a tyrosinase inhibitor in cosmetic and pharmaceutical formulations,^64^ can reach titers in the tens of grams per liter under optimized submerged fermentation, for example approx. 50 g L^−1^ in *Aspergillus oryzae* var. *effusus* NRC14 ^65^ and up to roughly 80 g L^−1^ in Aspergillus flavus ASU45 (OL314748).^66^ Even higher titers approaching 100 g L^−1^ have been reported in mutant *Aspergillus oryzae* strains.^67^ Nevertheless, the achieved productivity of strain ST006148 is sufficient to supply milligram quantities of Altenusin for downstream evaluation as an anti-tyrosinase compound.^64–67^

Therefore, 1.5 L optimized culture broth that has been obtained from 5 days fermentation with the bioreactor setup, was freeze-dried and the resulting solid was extracted with MeOH. The methanolic extract was concentrated to dryness under reduced pressure using a rotary evaporator, the residue was redissolved in water, and the aqueous solution was subjected to liquid–liquid extraction with ethyl acetate (aqueous phase : organic phase 1 : 2, v/v), which transferred the target metabolite into the organic phase. The ethyl acetate layer was concentrated under reduced pressure, and the resulting crude extract was dissolved in 5 mL of 40% (v/v) MeOH in H_2_O. This solution was subjected to HPLC purification (Fig. S4), affording a total of 0.1845 g of Altenusin with >95% purity from a calculated amount of 0.332 g in the crude extract (based on the bioreactor titer), corresponding to an overall recovery of around 80%.

The molecular formula of the purified compound was confirmed as C_15_H_14_O_6_ on the basis of a prominent ion peak at *m*/*z* = 291.0863 [M + H]^+^ in the HRMS (ESI) spectrum. Further elucidation and detailed analysis of 1D (^1^H (Fig. S5), ^13^C (Fig. S6)) and 2D NMR spectra (HMBC (Fig. S7), HSQC (Fig. S8)), together with comparison of the full NMR and MS data set (Table S4) with literature values, confirmed the structure as Altenusin,^68^ key HMBC correlations supporting this assignment are shown in Fig. S9.

### Anti-tyrosinase profiling of Altenusin

**Fig. 3:**
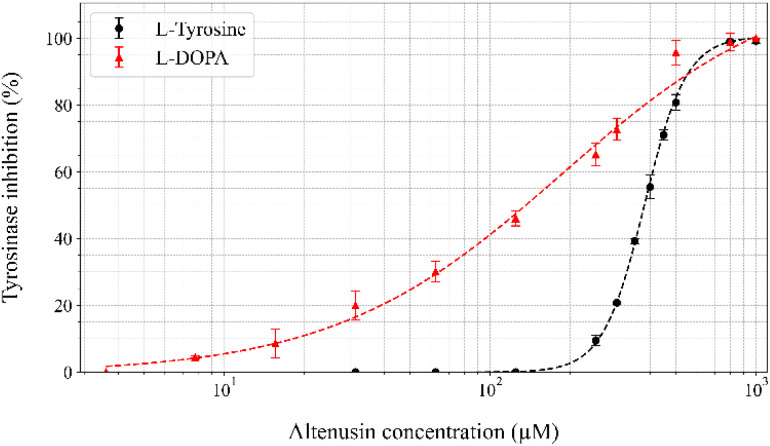
Dose-response curves showing the inhibitory effect of Altenusin on tyrosinase activity using l-tyrosine (● black) and l-DOPA (▲ red) as substrates. Enzyme inhibition was measured at various concentrations of Altenusin, and results are expressed as percentage inhibition relative to a control without inhibitor. Error bars represent standard deviation (*n* = 3).

With the structure established as Altenusin, we next assessed its inhibitory effect on mushroom tyrosinase by monitoring monophenolase and diphenolase activities with l-tyrosine and l-DOPA, respectively. Dose-response curves for mushroom tyrosinase revealed substrate-dependent inhibition (Fig. 3 and Table S5). With l-tyrosine, Altenusin did not cause detectable inhibition below 0.2 mM; at higher concentrations, the percentage of inhibition increased steeply, yielding IC_50_ of 0.381 ± 0.002 mM (Table S6). With l-DOPA, inhibition increased more gradually from low concentrations and exceeded 90% at nearly 0.8 mM, yielding an IC_50_ of 0.162 ± 0.023 mM, indicating more efficient inhibition of diphenolase than monophenolase activity.

This substrate-dependent behavior aligns with the distinct catalytic requirements and lag phenomena that differentiate monophenolase and diphenolase reactions in tyrosinases and with previous reports of substrate-dependent tyrosinase inhibition.^69,70^ Because apparent IC_50_ values are strongly influenced by assay format (substrate identity and concentration, enzyme source and loading), we next determined mechanism-linked steady-state parameters (*K*_i_, 
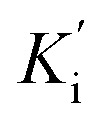
, Table S6) to provide a more robust basis for ranking Altenusin against other tyrosinase inhibitors.

**Fig. 4:**
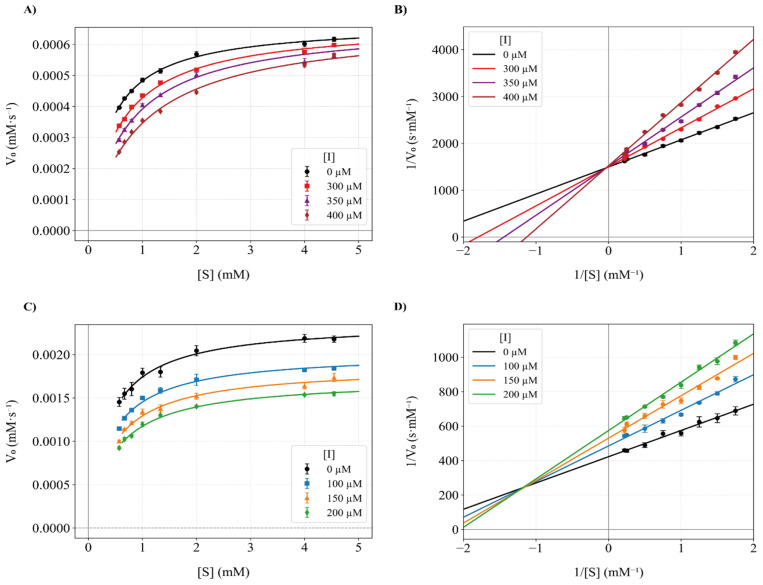
Steady-state kinetics of mushroom tyrosinase inhibition by Altenusin. (A and B) Monophenolase activity with l-tyrosine as substrate: (A) Michaelis–Menten plots of initial velocity (*V*_0_) *versus* [S] at 0, 300, 350 and 400 µM Altenusin; (B) corresponding Lineweaver–Burk plots (1/*V*_0_*versus* 1/[S]). (C and D) Diphenolase activity with l-DOPA as substrate: (C) Michaelis–Menten plots at 0, 100, 150 and 200 µM Altenusin; (D) corresponding Lineweaver–Burk plots. Symbols represent mean ± SD (*n* = 3).

In the monophenolase assay with l-tyrosine (Fig. 4A and B), Altenusin behaved as a competitive inhibitor. *V*_max_ remained essentially constant across 0–0.4 mM inhibitor, whereas *K*_m_ increased from 0.39 to 0.89 mM; global fitting gave *K*_i_ = 0.47 ± 0.19 mM (*α* = 1.86 ± 0.45, Table S7). In the diphenolase assay with l-DOPA (Fig. 4C and D), Altenusin produced a mixed-type inhibition pattern: *V*_max_ decreased from 2.37 × 10^−3^ to 1.74 × 10^−3^ mM s^−1^, while *K*_m_ rose from 0.36 to 0.49 mM. Mixed-model fitting yielded *K*_i_ = 0.253 ± 0.02 mM (*α* = 1.62 ± 0.24, free enzyme) and 
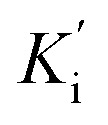
 = 0.61 ± 0.043 mM (*α*′ = 1.25 ± 0.11, ES complex) (Table S7).

Compared with well-characterized reference inhibitors, Altenusin ranks as a moderate inhibitor. Kojic acid typically shows IC_50_ values in the mid-tens of micromolar and mixed-type inhibition through dicopper chelation,^6,29,71,72^ making Altenusin approximately 1.3 to 6.2-fold less potent under comparable conditions. Within the alternariol family, dihydroaltenuene B reaches an IC_50_ of about 0.038 mM on mushroom tyrosinase,^73^ illustrating that modest modifications of the biphenyl-like core can yield several-fold gains in potency.

Previous studies have shown that alternariol and its structurally related mycotoxins exhibit *in vitro* cytotoxicity toward human hepatocytes (HepG2), followed by reactive oxygen species (ROS) formation and DNA damage.^74–76^ More broadly, redox-active phenolic scaffolds frequently exhibit narrow safety margins, particularly when they can both, donate electrons and interact with transition metals.^77,78^ Motivated by this apparent coupling between tyrosinase-directed activity and cytotoxic risk in the alternariol family, we next assessed the cytotoxicity of Altenusin by measuring 48 h viability of human HepG2 cells using a WST-1 assay. HepG2 cells are widely used as a first-pass human liver-derived model for toxicology and genotoxicity-oriented screening, offering strong comparability to published datasets.^79,80^ Moreover, cytotoxicity profiling supports dose selection for standard genotoxicity assays (*e.g.*, *in vitro* micronucleus testing, OECD TG 487), which represent an early step in safety assessment for both cosmetic and food-related applications.^81,82^

Altenusin reduced HepG2 viability in a concentration-dependent manner, yielding a CC_50_ of 0.093 mM (Fig. S10). To position cytotoxicity relative to target inhibition, we compared the cellular CC_50_ to our *in vitro* potencies of the l-DOPA assay (IC_50_ = 0.162 mM) and the l-tyrosine assay (IC_50_ = 0.381 mM), both revealing selectivity indices (SI) < 1 (SI_l-DOPA_ = 0.58 and SI_l-tyrosine_ = 0.24 (CC_50_/IC_50_)). In comparison, kojic acid shows low intrinsic HepG2 cytotoxicity with reported IC_50_ values around 8.02 mM.^83^ However, quercetin, a plant flavonoid, that finds application as antioxidant in cosmetic products exhibits cytotoxicity against HepG2 cells with IC_50_ values of 0.05 mM.^84^ This has also been demonstrated in WST-1 proliferation assays revealing reduced metabolic activity of HepG2 cells within 24–48 h in presence of quercetin at tens-of-micromolar concentrations (up to 0.08 mM).^85^ Thus, Altenusin appears substantially more cytotoxic than kojic acid; however, falls into a broadly similar HepG2 cytotoxicity range as quercetin, while acknowledging the differences between MTT and WST-1 readouts. In summary, our results classify Altenusin as a moderately potent carboxy-biphenyl scaffold for tyrosinase inhibition, whose narrow selectivity indices towards HepG2 cells illustrating the close relationship between activity and toxicity in this chemotype. Resorcinol-type motifs and related phenolic patterns are repeatedly reported for potent tyrosinase inhibition, including for human tyrosinase-directed inhibitor series, supporting systematic tuning of the phenolic substitution pattern in Altenusin (*e.g.*, stepwise *O*-alkylation/*O*-acylation or demethylation to probe the contribution of each OH group to binding at the dicopper site).^86^

### Direct binding and thermodynamic analysis by fluorescence quenching

**Fig. 5:**
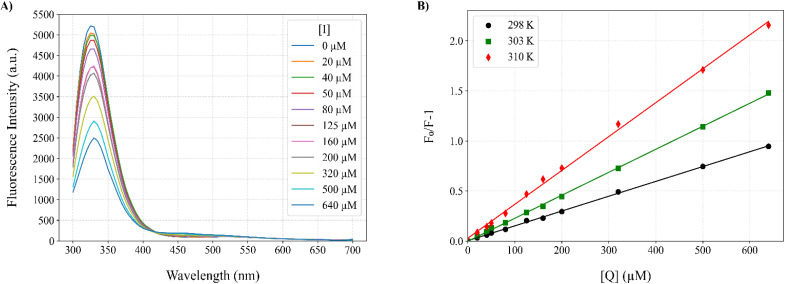
Fluorescence quenching of mushroom tyrosinase by Altenusin. (A) Intrinsic fluorescence emission spectra of tyrosinase in the absence and presence of increasing concentrations of Altenusin ([I] = 0–640 µM) at 298 K. (B) Stern–Volmer plots of (*F*_0_/*F* − 1) *versus* Altenusin concentration at 298, 303, and 310 K, showing a linear dependence consistent with mixed quenching behavior.

Having defined its activity–toxicity profile, we next probed the direct interaction of Altenusin with mushroom tyrosinase by monitoring the intrinsic fluorescence of the enzyme upon titration with the inhibitor and analyzed the resulting quenching profiles to derive binding stoichiometry and thermodynamic parameters. Increasing inhibitor concentrations (0–640 µM) led to a progressive, temperature-dependent decrease in the intrinsic emission of the enzyme (*λ*_ex_ = 280 nm, *λ*_em_ = 300–700 nm), with marked quenching of the Trp-dominated maximum at 330–350 nm at 298 K (Fig. 5A). Comparable spectra recorded at 303 and 310 K showed the same qualitative behavior (Fig. S11). Stern–Volmer plots were essentially linear at all three temperatures (Fig. 5B) and yielded *K*_SV_ values of 1.48 × 10^3^, 2.31 × 10^3^ and 3.39 × 10^3^ M^−1^ at 298, 303 and 310 K, respectively (Table S8).

Assuming a typical lifetime for protein fluorescence (*τ*_0_ = 10^−8^ s),^87–89^ the corresponding bimolecular quenching constants *K*_q_ = *K*_SV_/*τ*_0_ fall in the 10^11^ M^−1^ s^−1^ range, well above the diffusion-controlled limit (About 10^9^–10^10^ M^−1^ s^−1^) (Table S8). Although these *K*_q_ values are only approximate, because *τ*_0_ was not measured directly for this tyrosinase preparation, they robustly indicate a substantial static contribution to quenching. In contrast, the increase in *K*_SV_ with temperature is more typical of collisional quenching. Together, these observations are most consistent with a mixed static/dynamic mechanism, rather than a purely collisional one.^90^ Modified Stern–Volmer (double-log) analysis gave apparent site numbers close to unity (0.96–0.98) and association constants *K*_a_ of 1.64 × 10^3^, 2.56 × 10^3^ and 4.44 × 10^3^ M^−1^ at 298, 303 and 310 K, respectively (Table 1). Thereby indicating a single predominant binding site and modest affinity (*K*_d_ ≈ 0.2–0.6 mM). Thus, fluorescence quenching supports direct complex formation between Altenusin and tyrosinase, with interaction strengths that are consistent with the mid-micromolar IC_50_ values obtained from steady-state kinetics.

**Table 1:** Binding and thermodynamic parameters for the Altenusin-tyrosinase interaction derived from fluorescence quenching

*T* (K)	*K*_a_ (M^−1^)^a^	*K*_d_ (mM)^b^	*N* a	Δ*G*° (kJ mol^−1^)^c^
298	1.64 × 10^3^	0.61	0.98	−18.4
303	2.56 × 10^3^	0.39	0.98	−19.7
310	4.44 × 10^3^	0.23	0.96	−21.7

a Association constants (*K*_a_) and apparent binding stoichiometries (*n*) were obtained from modified Stern–Volmer (double-log) plots.

b *K*_d_ was calculated as *K*_a_^−1^.

c Δ*G*° values were derived from Δ*G*° = −*RT* ln *K*_a_.

The temperature dependence of *K*_a_ enabled a Van't Hoff analysis (Fig. S11), which yielded a positive enthalpy change (Δ*H*° = +63.62 kJ mol^−1^) and a large positive entropy change (Δ*S*° = +160.22 J mol^−1^ K^−1^), resulting in favorable binding free energies (Δ*G*°) that became more negative at higher temperature (Δ*G*° = −18.4, −19.7 and −21.7 kJ mol^−1^ at 298, 303 and 310 K, respectively; Table 1). This (Δ*H*° > 0, Δ*S*° > 0) signature is characteristic of entropy-driven binding dominated by desolvation and hydrophobic contacts, rather than the exothermic, enthalpy-driven complexes typically associated with hydrogen-bond and van der Waals domination.^90,91^

In the context of our kinetic data, these thermodynamic features suggest that Altenusin engages a relatively hydrophobic region of the enzyme through an interaction that is strengthened at elevated temperature but remains reversible and of moderate affinity. Compared with canonical natural inhibitors of tyrosinase, Altenusin occupies a distinct mechanistic space. Kojic acid, for example, has been reported to form a much tighter complex (*K*_a_ close to 10^5^ M^−1^ at 298 K) and to quench tyrosinase fluorescence *via* a predominantly static mechanism, with *K*_SV_ decreasing as temperature increases, and thermodynamic analyses indicate binding mainly driven by hydrogen bonds and van der Waals interactions, a profile typically associated with exothermic, enthalpy-driven complexes (Δ*H*° < 0, Δ*S*° < 0).^92^ Several flavonoid inhibitors similarly exhibit higher affinities (*K*_a_ around 10^4^–10^5^ M^−1^) and mainly static quenching behavior.^93,94^ Relative to these benchmarks, Altenusin forms a weaker but thermodynamically distinct complex, characterized by entropy-driven, hydrophobically assisted binding and mixed static/dynamic quenching. This divergence from kojic acid and flavonoids suggests that the carboxy-biphenyl scaffold binds to a distinct, more hydrophobic microenvironment in the tyrosinase active site and highlights specific features such as the redox-active biphenyl core and the balance between hydrophobic and hydrogen-bonding contacts.

### Evaluation of copper-chelating/reducing and DPPH/ABTS scavenging

Given the redox-active nature of the biphenyl core and the dicopper architecture of tyrosinase, we next evaluated the radical-scavenging and copper-interaction properties of Altenusin to assess whether redox and metal-based mechanisms contribute to its overall inhibitory profile. Therefore, we conducted a series of *in vitro* assays quantifying copper chelation (using pyrocatechol violet, PV), copper reduction (Cu^2+^ to Cu^+^) using bathocuproine disulfonic acid (BCS) as well as DPPH and ABTS radical scavenging.

In the PV-based copper chelation assay (Fig. S12A and Table S9), Altenusin exhibited an IC_50_ of 0.906 ± 0.043 mM, closely matching the values reported for monomeric flavonoids such as (+)-catechin (IC_50_: 0.890 mM), epicatechin (1.11 mM) and resveratrol (0.937 mM) under comparable conditions.^95^ In contrast, the synthetic chelator EDTA, used here as a positive control, exhibited an IC_50_ of 0.0162 ± 0.0002 mM, consistent with its high-affinity metal-binding behavior. Its moderate binding affinity likely arises from its phenolic –OH groups coordinating in non-ideal geometries: predominantly as monodentate ligands *via* individual hydroxyl donors, analogous to flavonol coordination through the 3-OH group in tyrosinase active-site models,^96^ with only occasional weak bidentate bridging of its catechol pattern between copper active center.^97^ Nonetheless, this level of Cu^2+^ coordination by Altenusin might be sufficient to inhibit copper-dependent enzymes: numerous phenolic and polyphenolic compounds are known to inhibit tyrosinase through metal chelation.^5^ Bioinspired catecholic frameworks demonstrate that even moderate Cu^2+^ binding can effectively attenuate metalloenzyme activity.^8^

In the copper-reducing assay (Fig. S12B and Table S9) employing bathocuproine disulfonic acid (BCS) as the Cu^+^-selective chromogenic agent (CUPRAC-BCS format), we determined the IC_50_ of Altenusin to be 0.2598 ± 0.0016 mM and that of l-ascorbic acid to be 0.1648 ± 0.0011 mM under identical conditions. Thus, Altenusin is approximately 1.6-fold less potent than ascorbic acid in this assay. Although neocuproine-based CUPRAC assays remain the most common means to assess copper-reducing power, the BCS variant provides superior selectivity for the Cu^+^ oxidation state and minimizes interference from Cu^2+^ residual.^98^ Quantitative IC_50_ values for natural products in BCS-based reduction assays are notably scarce. Most studies address total antioxidant capacity in complex mixtures rather than defined compounds. To date, only a few simple flavonoids and phenolic acids have been characterized,^48,95^ making our data a valuable first carboxy-biphenyl benchmark compound for future comparison on electron-transfer efficacy and guiding structure-activity as well as mechanistic studies.

In the DPPH assay (Fig. S12C and Table S9), Altenusin exhibited an IC_50_ of 0.0317 ± 0.0020 mM, marginally more potent than the positive control – Trolox (0.0361 ± 0.0022 mM, under identical conditions) corresponding to a Trolox-equivalent (TEAC) activity of approx. 1.1, that is, Altenusin is 10 to 15% more potent than Trolox under our assay conditions. This activity, attributable to the resonance stabilization of its phenoxyl radicals *via* HAT/SET pathways^99–101^ and reinforced by its catechol moiety on ring B (4′,5′-dihydroxyl substitution) together with an extended conjugated aromatic system,^102^ also surpasses kojic acid (IC_50_: 0.7 mM ^103^) and falls within the range reported for resveratrol (0.060 to 0.085 mM ^104,105^). Although catechol-bearing polyphenols such as quercetin (IC_50_: 0.018 mM ^106^) still demonstrate higher radical-scavenging potency, the combination of the catechol motif and conjugation in Altenusin provides a compelling balance of activity. Nevertheless, because DPPH protocols can vary between studies (*e.g.*, solvent, radical concentration, incubation time), future electrochemical and rapid-kinetics investigations will be essential to elucidate the predominant mechanism and confirm the biological relevance of these antioxidant properties. In the ABTS assay (Fig. S12D and Table S9), Altenusin also showed strong radical-scavenging activity, with an IC_50_ of 0.0663 ± 0.0015 mM compared with 0.0221 ± 0.0001 mM for Trolox under identical conditions. This corresponds to a TE of 0.33, indicating that Altenusin remains active but is roughly threefold less potent than Trolox in the ABTS system. Together, the DPPH and ABTS data demonstrate that Altenusin behaves as a robust, assay-dependent antioxidant.

Altogether, these data show that Altenusin combines a potent DPPH/ABTS radical-scavenging capacity (TEAC = 1.1 and 0.33, respectively) with an intermediate copper-reducing enzymatic activity (IC_50_ BCS = 0.2598 ± 0.0016 mM) while showing moderately low Cu^2+^ binding affinity (IC_50_ = 0.906 ± 0.043 mM). In the context of tyrosinase, this combination of radical scavenging, copper reduction and modest Cu^2+^ chelation illustrate how carboxy-biphenyl scaffolds can modulate dicopper oxidases through conjugated binding and redox mechanisms, rather than through high-affinity metal sequestration alone. Future investigations using isothermal titration calorimetry, speciation-sensitive spectroscopy, and direct enzymatic assays will be essential to elucidate the thermodynamic and kinetic details of its interaction with Cu^2+^ and its impact on copper-dependent enzymes.

## Conclusions

In this work, we combined cheminformatics, bioprocess engineering and biochemical characterization to establish the fungal biphenyl polyketide Altenusin as a mechanistically defined and fermentatively accessible scaffold for tyrosinase inhibition. A structure-based screen of a microbial derived natural product library, followed by docking to AbPPO_3_, identified Altenusin as a plausible binder of the dicopper active site. Optimization of submerged fermentation by OSMAC and Box–Behnken design, together with transfer to a DO-controlled stirred-tank bioreactor, increased Altenusin titers into the approximately 0.25 g L^−1^, sufficient to support milligram-scale supply from a non-engineered fungus under well-defined, scalable conditions.

At the enzymatic level, Altenusin acts as a substrate-dependent inhibitor of mushroom tyrosinase, displaying competitive inhibition of monophenolase activity and mixed-type inhibition of diphenolase activity with mid-micromolar potencies. Fluorescence quenching and Van't Hoff analysis indicate single-site, entropy-driven binding with a mixed static/dynamic component, consistent with hydrophobically assisted engagement of the active site pocket. Complementary redox profiling shows that Altenusin combines strong radical-scavenging capacity and copper-reducing activity with low Cu^2+^ chelation. This suggests that it can modulate copper enzymes through a combination of direct binding and redox effects, rather than by high-affinity metal sequestration alone.

From a medicinal-chemistry perspective, Altenusin provides a tractable carboxy-biphenyl starting point for a focused SAR campaign. In parallel, cytotoxicity assays in HepG2 cells revealed that the concentrations required for robust tyrosinase inhibition partially overlap with those that reduce cellular viability, yielding narrow selectivity indices and arguing for caution in their further investigation towards *e.g.*, cosmetic or food-preservative applications. Together with its moderately potent activity Altenusin might not be regarded as a ready-to-use tyrosinase inhibitor, but rather as a biotechnologically and chemically tractable starting point within the intriguing carboxy-biphenyl family. More broadly, the combined enzymatic, redox and cytotoxicity data begin to delineate design limitations for future carboxy-biphenyl derivatives with improved activity–liability profiles.

## Author contributions

N. R. C., M. S. and T. F. S. drafted the manuscript. N. R. C., M. S., M. M. and T. F. S. designed the study. N. R. C., M. S., C. M. Z., Y. L., M. A. P., C. K., F. M. and A. G. performed the experiments.

## Conflicts of interest

The authors report no conflicts of interest.

## Supplementary Material

RA-016-D5RA09904H-s001

## Data Availability

The data supporting the findings of this study are included within the article and its supplementary information (SI). Additional data are available from the corresponding author upon reasonable request. Supplementary information: upstream/downstream processing details, additional mechanistic assay datasets and curve fits, and supporting analytical characterization. See DOI: https://doi.org/10.1039/d5ra09904h.
