# Supplementary material for: Mechanistic profiling and optimized production of Altenusin, a fungal carboxy-biphenyl scaffold for tyrosinase inhibition
Source: RSC Adv. 2026 Feb 19;16(11):9834–50. doi: 10.1039/d5ra09904h (PMC12917734; doi:10.1039/d5ra09904h)
Supplement: RA-016-D5RA09904H-s001 [file RA-016-D5RA09904H-s001.pdf]

Supplementary Information (SI) for RSC Advances.
This journal is © The Royal Society of Chemistry 2026

Supplementary Information

Mechanistic profiling and optimized production of Altenusin, a fungal carboxy-biphenyl scaffold for tyrosinase inhibition

Nicolas Reyes Castillo[a,b], Marius Spohn*[b], Celine M. Zumkeller[a,b], Michael Marner[b], Yang Liu[a,b], Maria Patras[a,b], Christian Kersten[c,d], Francesca Magari[e], Arnold Grünweller[e] and Till. F. Schäberle*[a,b,f].

a Natural Product Research, Institute for Insect Biotechnology, Justus-Liebig-University Giessen, 35392 Giessen, Germany.

b Fraunhofer Institute for Molecular Biology and Applied Ecology, Branch of Bioresources, Ohlebergsweg 12, 35392 Giessen, Germany.

c Institute of Pharmaceutical and Biomedical Sciences, Johannes Gutenberg-University Mainz, Staudinger Weg 5, 55128 Mainz, Germany.

d Institute for Quantitative and Computational Bioscience, Johannes Gutenberg-University Mainz, BioZentrum l, Hanns-Dieter-Hüsch Weg 15, 55128 Mainz, Germany.

e Institute of Pharmaceutical Chemistry, Philipps-University Marburg, Marbacher Weg 6, 35032 Marburg, Germany

f German Center for Infection Research (DZIF), Partner Site Giessen-Marburg - Langen, Ohlebergsweg 12, 35392 Giessen, Germany.

Table of Contents

Fig. S1. CORASON comparison of biosynthetic gene clusters (BGCs) related to region 1 (T1PKS). ...3

Fig. S2. Parity plot of predicted versus experimental Altenusin production for the four-factor Box–Behnken response surface model in shake flasks. 3

Fig. S3. Box–Behnken response surfaces for Altenusin in shake flasks. 4

Fig. S4. HPLC purification of Altenusin from the ethyl acetate extract of the optimized 1.5 L bioreactor culture of strain ST006148. 4

Fig. S5. ^{1}H NMR spectrum of Altenusin used for structure elucidation (see Table S4). 5

Fig. S6. ^{13}C NMR spectrum of Altenusin used for structure elucidation (see Table S4). 5

Fig. S7. HMBC spectrum of Altenusin showing key long-range ^{1}H-^{13}C correlations (see Table S4). 6

Fig. S8. HSQC spectrum of Altenusin showing direct ^{1}H-^{13}C correlations (see Table S4). 6

Fig. S9. Key HMBC (^{1}H-^{13}C) correlations observed for Altenusin, mapped onto its chemical structure. 7

Fig. S10. Cytotoxicity results. 7

Fig. S11. Altenusin quenches tyrosinase fluorescence in a concentration- and temperature-dependent manner. 7

Fig. S12. Dose–response activity of Altenusin in antioxidant and metal chelation assays. 8

Fig. S13. A) Re-docking pose of tropolone (green carbon atoms, RMSD = 2.1 Å, HYDE-score: -28.3 kJ/mol) in complex with AbPPO3 (white carbon atoms and surface, PDB-ID: 2Y9X). 8

Fig. S14. Molecular docking predicted binding modes (green carbon atoms) and predicted affinities (HYDE scores) of known PPO inhibitors....9

Table S1. Box–Behnken design matrix for Altenusin production in shake flasks. 10

Table S2. ANOVA for the quadratic RSM model (Box–Behnken). 11

Table S3. Model summary statistics and lack-of-fit tests for sequential model building (linear, 2FI, quadratic, cubic). 11

Table S4. NMR spectroscopic data of Altenusin (^{1}H NMR in 700 MHz, ^{13}C NMR in 176 MHz, δ in ppm; DMSO-d_6)....12

Table S5. Nonlinear regression parameters for Altenusin inhibition of mushroom tyrosinase using L-tyrosine and L-DOPA as substrates. 12

Table S6. Michaelis–Menten kinetic parameters for mushroom tyrosinase in the absence and presence of Altenusin. 13

Table S7. Lineweaver–Burk fits and competitive/mixed-inhibition parameters for Altenusin inhibition of mushroom tyrosinase (L-tyrosine and L-DOPA as substrate). 14

Table S8. Stern–Volmer and modified Stern–Volmer parameters for the interaction between Altenusin and mushroom tyrosinase. 15

Table S9. Nonlinear regression parameters for Altenusin and reference compounds in copper-chelation, copper(II)-reduction and radical-scavenging assays. 15

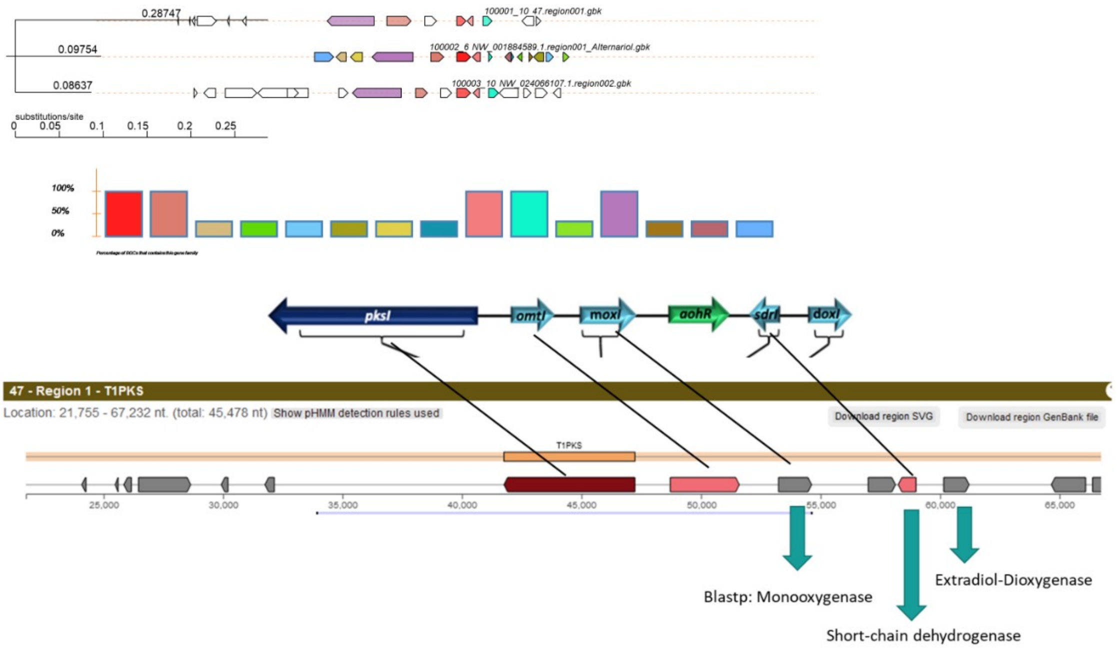

Fig. S1. CORASON comparison of biosynthetic gene clusters (BGCs) related to region 1 (T1PKS). The CORASON-derived core-gene phylogeny of the closest cluster (Alternariol), together with per-gene amino-acid identity (top). The antiSMASH result highlights the core pksI and adjacent tailoring genes (*omtI*, *moxI*, *aohR*, *sdrI*, *doxI*); selected ORFs were annotated by BLASTp (monooxygenase, short-chain dehydrogenase, and extradiol dioxygenase).

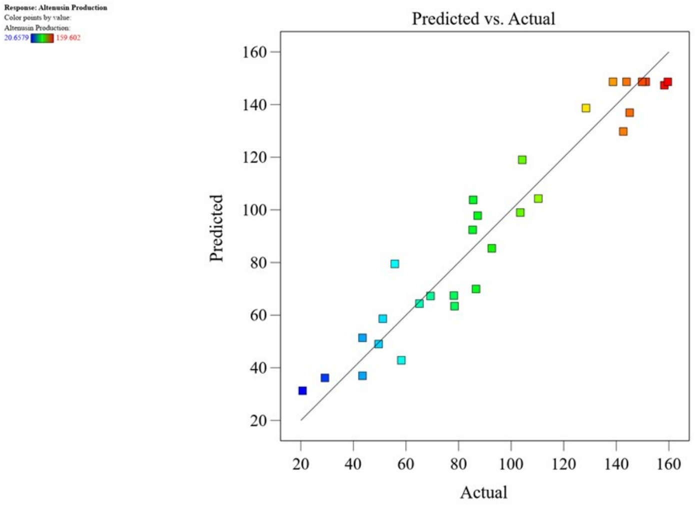

Fig. S2. Parity plot of predicted versus experimental Altenusin production for the four-factor Box–Behnken response surface model in shake flasks. Points correspond to individual design runs, colored by measured Altenusin titer, and the solid line represents perfect agreement (y = x), illustrating the good fit of the model.

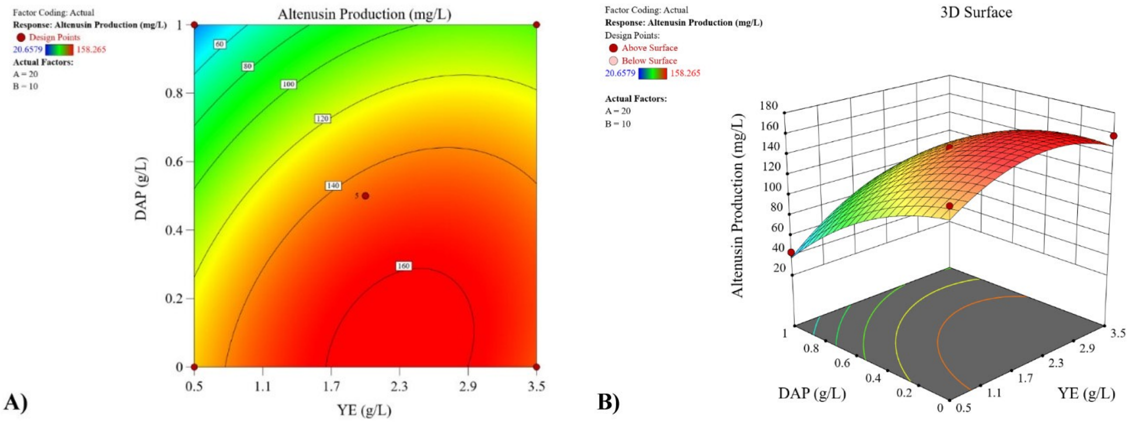

Fig. S3. Box–Behnken response surfaces for Altenusin in shake flasks. (A) Contour map of Altenusin (mg/L) as a function of **YE** and **DAP** at **ME = 20 g/L** and **G = 10 g/L**; circles denote experimental design points; shading and contour labels show model-predicted titers. (B) Corresponding 3D surface. The desirability-based optimum occurs at YE approx. 2.06 g/L, DAP around 0.27 g/L.

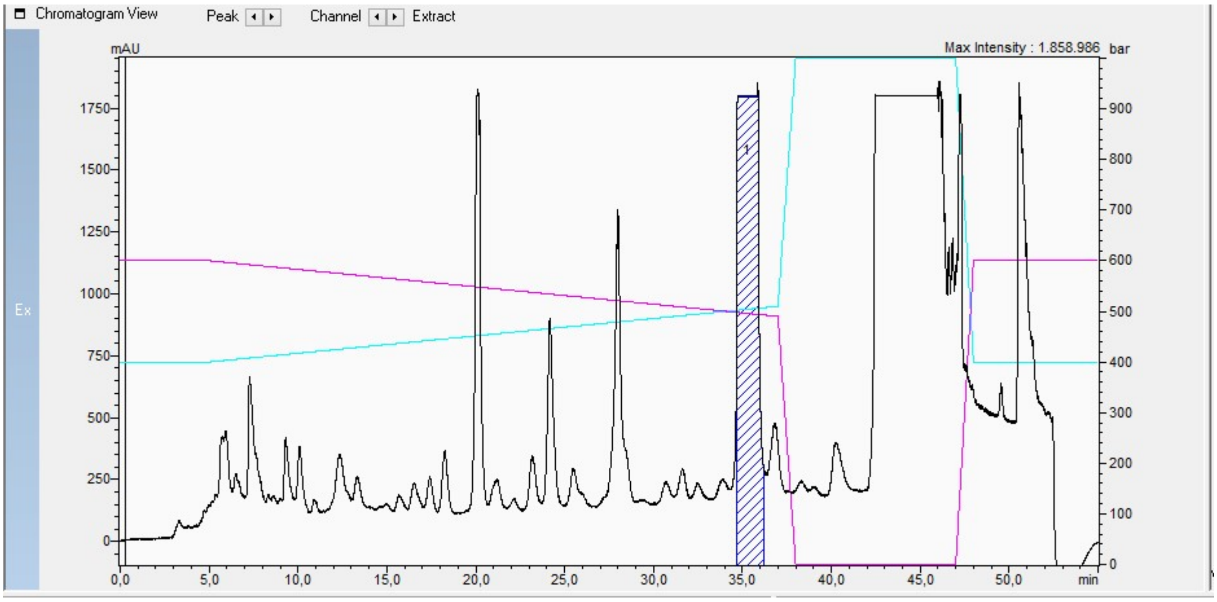

Fig. S4. HPLC purification of Altenusin from the ethyl acetate extract of the optimized 1.5 L bioreactor culture of strain ST006148. The black trace shows the UV chromatogram (mAU) recorded at 210 nm, and the blue hatched region indicates the retention window used to collect the Altenusin fraction. Pooled fractions yielded 0.1845 g of Altenusin at >95% purity (approx. 80% recovery based on the bioreactor titer); colored lines represent the solvent gradient and system parameters.

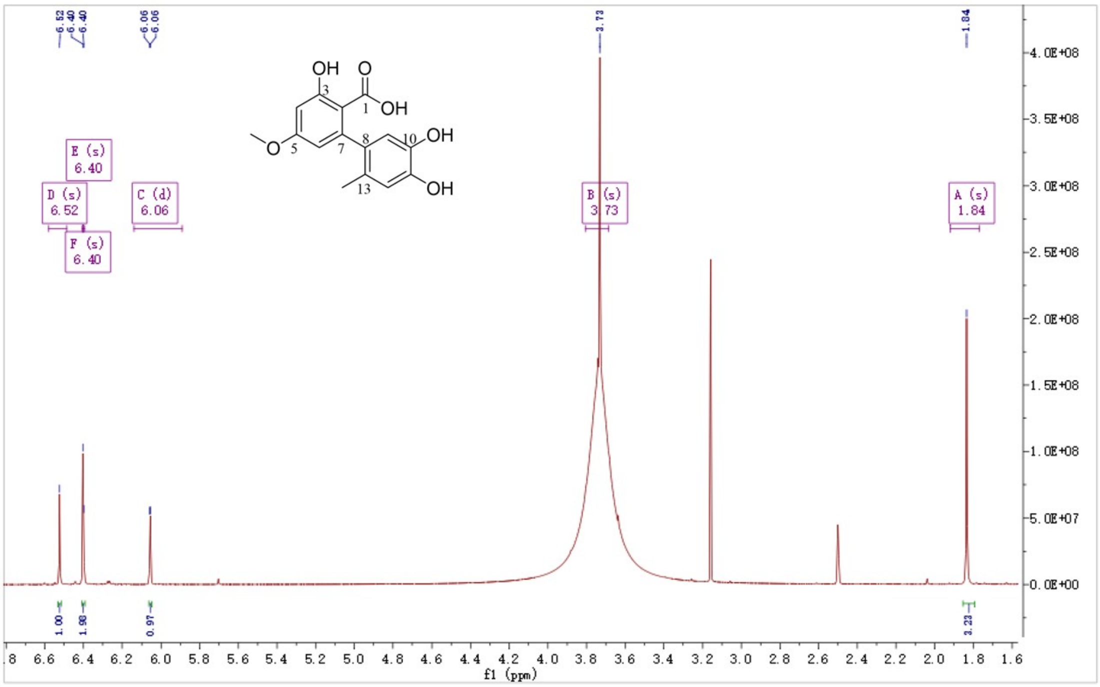

Fig. S5. ^{1}H NMR spectrum of Altenusin used for structure elucidation (see Table S4).

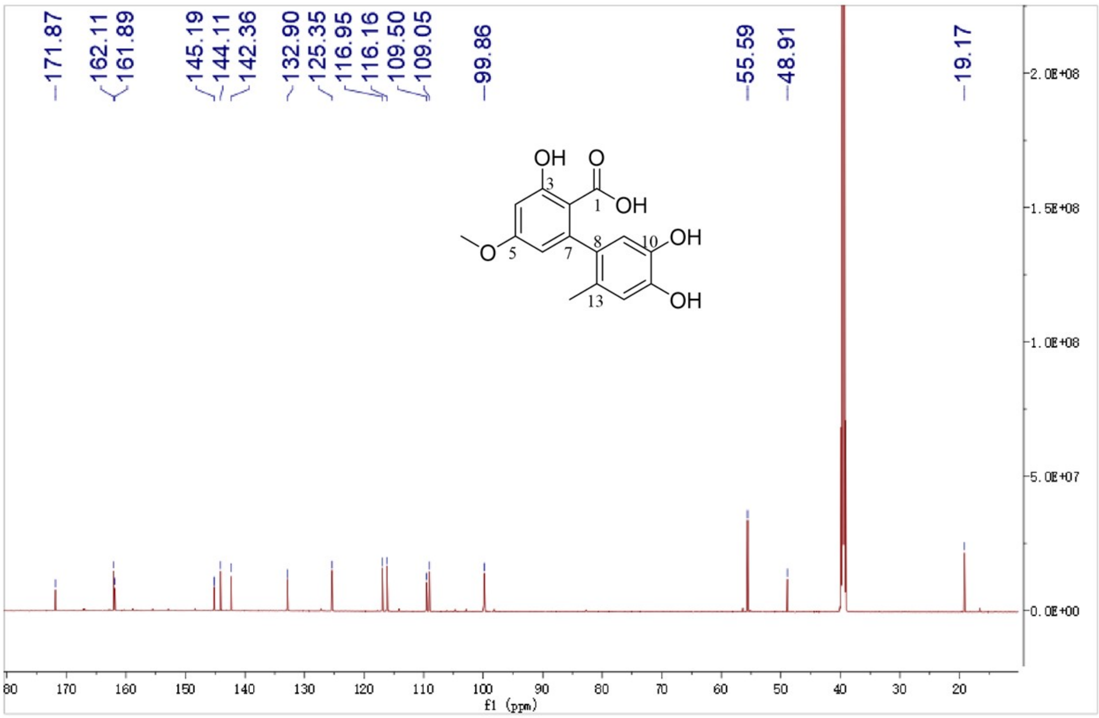

Fig. S6. ^{13}C NMR spectrum of Altenusin used for structure elucidation (see Table S4).

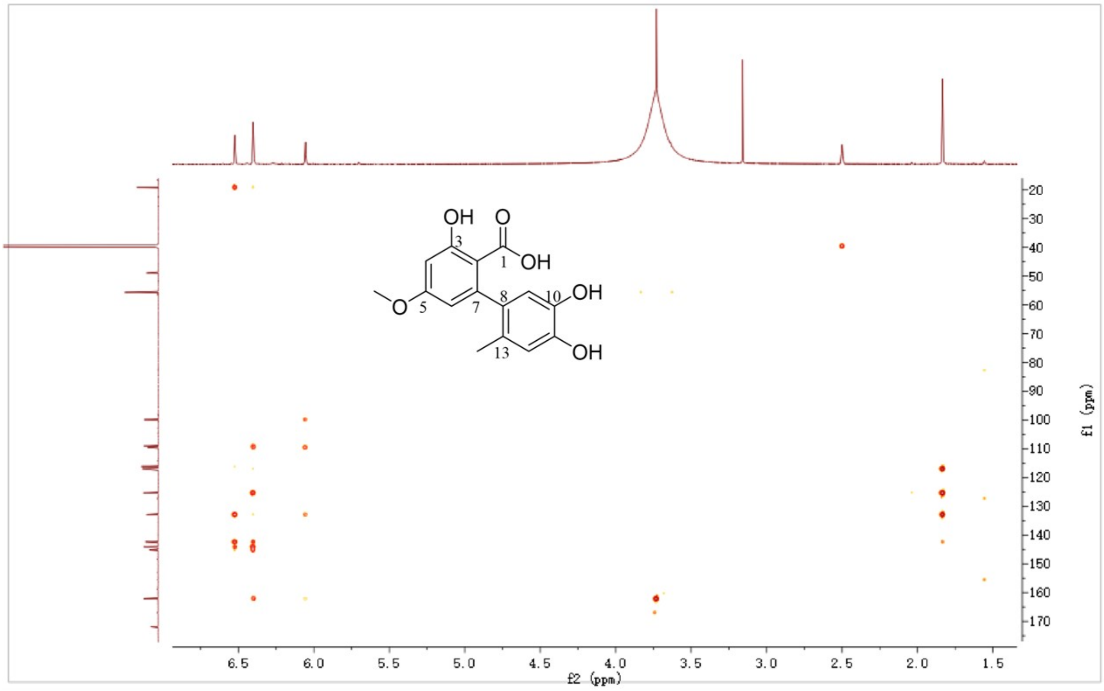

Fig. S7. HMBC spectrum of Altenusin showing key long-range ^{1}H-^{13}C correlations (see Table S4).

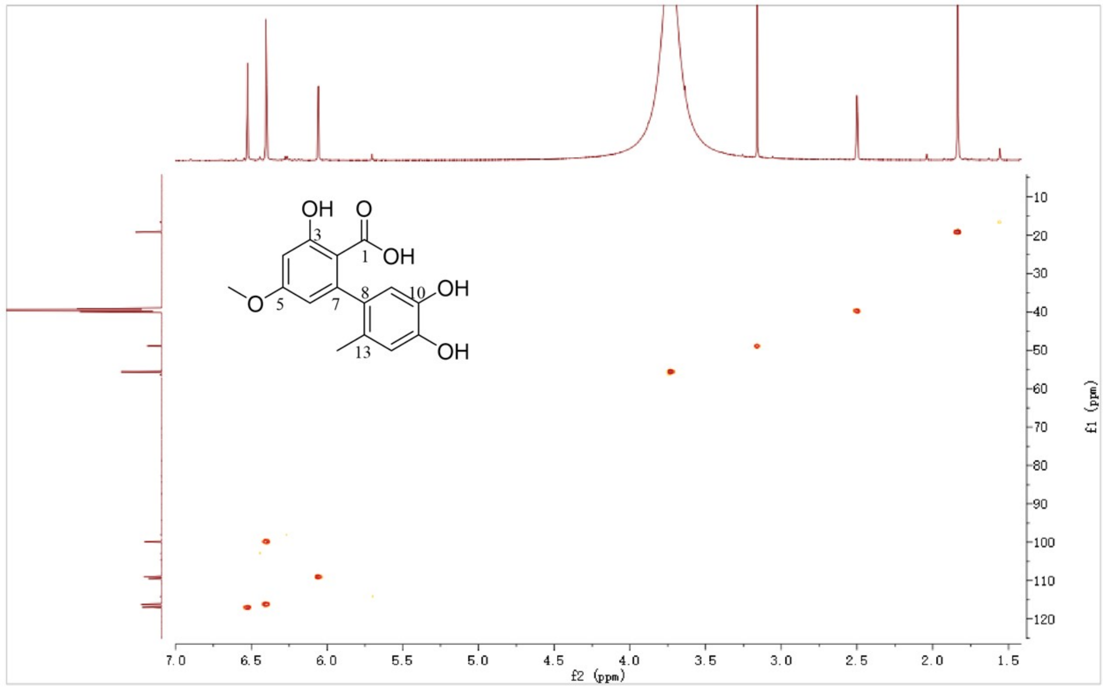

Fig. S8. HSQC spectrum of Altenusin showing direct ^{1}H-^{13}C correlations (see Table S4).

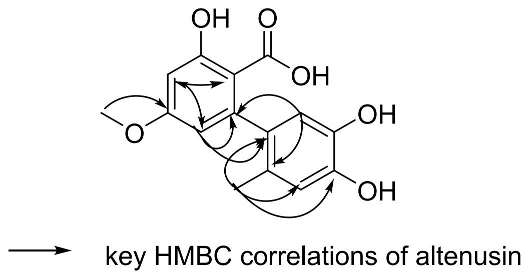

Fig. S9. Key HMBC (^1H-^{13}C) correlations observed for Altenusin, mapped onto its chemical structure.

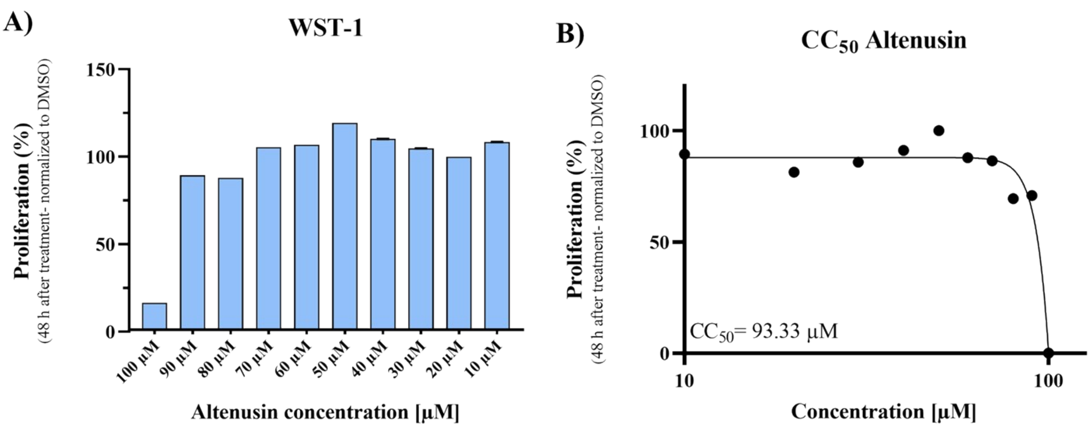

Fig. S10. Cytotoxicity results. A-B) HepG2 cells were incubated with the indicated concentrations of the compounds and WST-1 assays were performed after 48 h of incubation. Reduction in cell proliferation was calculated in percentage after normalization to DMSO control. The CC50 value was calculated using non-linear regression analysis resulting in a value of 93.33 µM. Standard error of the mean (SEM) was calculated for n ≥ 3.

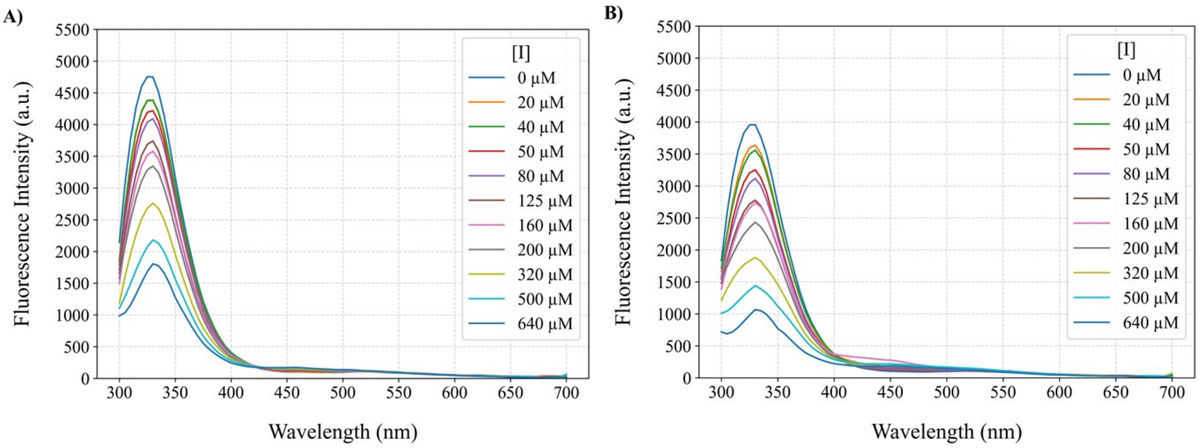

Fig. S11. Altenusin quenches tyrosinase fluorescence in a concentration- and temperature-dependent manner. Panel A) Emission spectra (λex = 280 nm; λem 300–700 nm) recorded at 303 K, Panel B) Emission spectra (λex = 280 nm; λem 300–700 nm) recorded at 310 K, upon addition of Altenusin ([Q] = 0–640 µM).

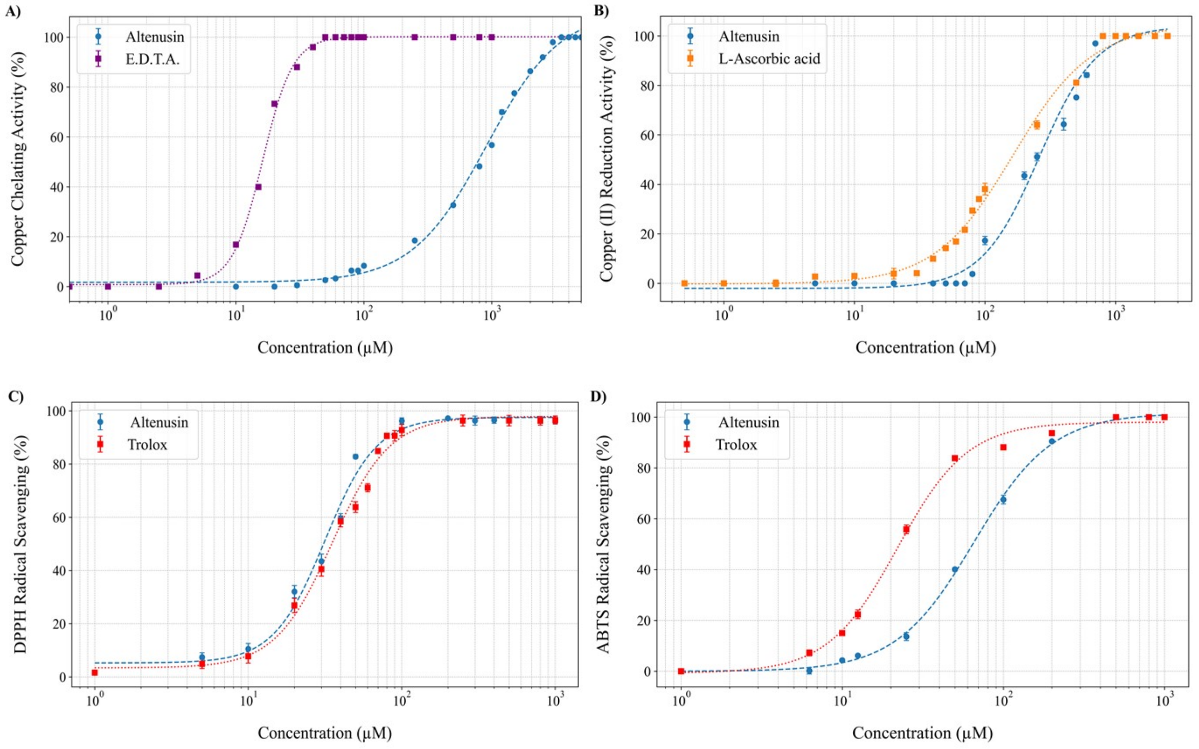

Fig. S12. Dose–response activity of Altenusin in antioxidant and metal chelation assays. (A) Copper chelating activity using EDTA as positive control. (B) Copper (II) reduction assay using L-ascorbic acid as positive control. (C) DPPH radical scavenging activity. (D) ABTS Radical scavenging activity. Data represent mean values ± standard deviation from three independent experiments.

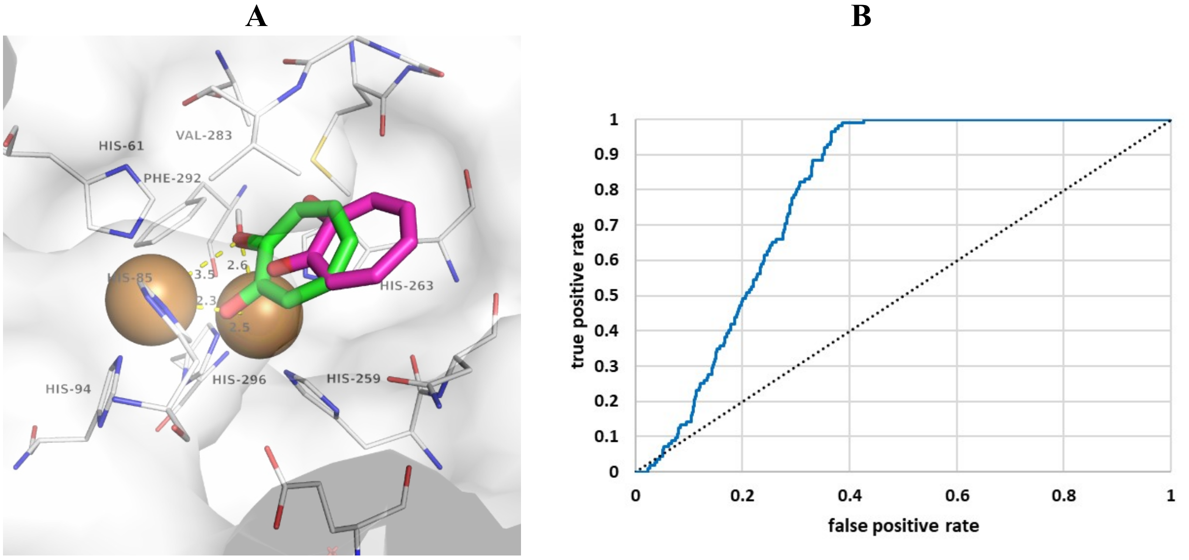

Fig. S13. A) Re-docking pose of tropolone (green carbon atoms, RMSD = 2.1 Å, HYDE-score: -28.3 kJ/mol) in complex with AbPPO3 (white carbon atoms and surface, PDB-ID: 2Y9X). Crystallographic binding mode of tropolone is shown with magenta-colored carbon atoms. Copper-complexation is depicted as yellow dashed lines with distances in Å. **B)** Binder-decoy discrimination receiver operating characteristics (ROC) curve. Area under the curve (AUC) of 0.79 indicates strong discrimination of binders from decoys (dotted line depicts random distribution with ROC AUC of 0.5).

A **B**

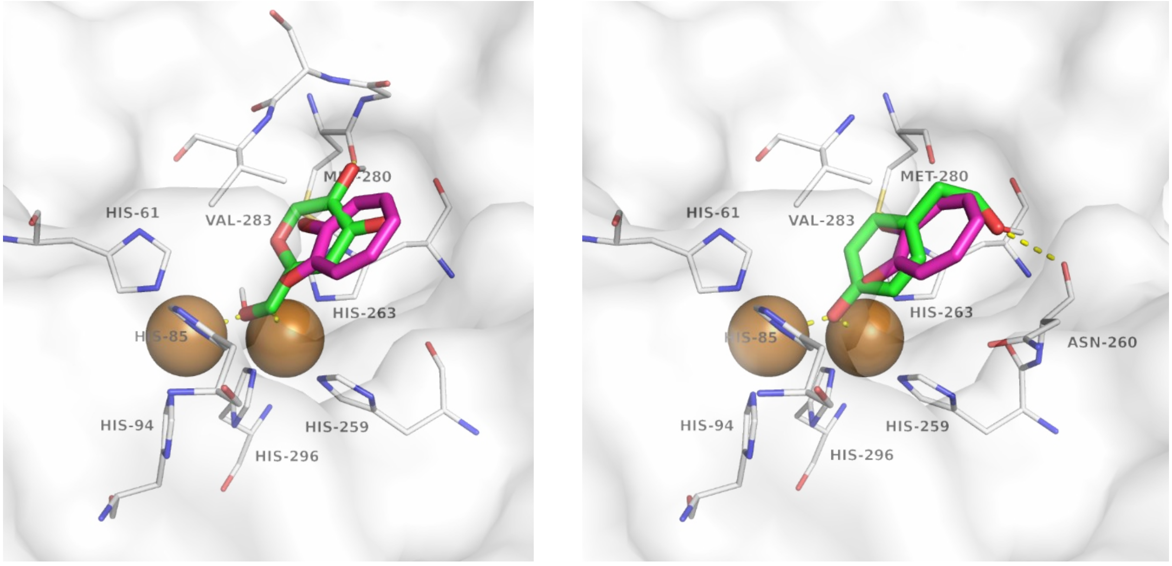

HYDE-score: -45.1 kJ/mol HYDE-score: -28.7 kJ/mol

Fig. S14. Molecular docking predicted binding modes (green carbon atoms) and predicted affinities (HYDE scores) of known PPO inhibitors. A) Kojic acid and B) p-tyrosinol in complex with *Ab*PPO3 (PDB-ID: 2Y9X, white carbon atoms and surface). Polar interactions are depicted as yellow dotted lines, while residues forming hydrophobic contacts or complexing the catalytic copper ions are also labelled. The crystallographic reference ligand tropolone is shown with magenta-colored carbon atoms for orientation.

Table S1. Box–Behnken design matrix for Altenusin production in shake flasks. Coded and actual factor levels for malt extract (ME), glucose (G), yeast extract (YE) and diammonium phosphate (DAP), together with the experimental Altenusin titers used to fit the response surface model.

**Run**	**Malt Extract (g/L)**	**$(NH_4)_2HPO_4$ (g/L)**	**Glucose (g/L)**	**Yeast Extract (g/L)**	**Altenusin production (mg/L)**
**1**	5	0.5	2.5	2	49.57
**2**	20	0.5	10	2	143.88
**3**	20	0.5	10	2	149.82
**4**	20	2	2.5	2	29.15
**5**	5	0.5	10	0.5	104.22
**6**	20	0.125	10	8	20.66
**7**	80	2	10	2	86.62
**8**	20	0.5	40	8	103.49
**9**	20	2	40	2	87.27
**10**	20	0.125	2.5	2	78.22
**11**	5	2	10	2	58.24
**12**	20	0.125	40	2	92.65
**13**	20	2	10	0.5	85.37
**14**	20	2	10	8	51.17
**15**	20	0.5	10	2	151.19
**16**	80	0.125	10	2	69.32
**17**	20	0.125	10	0.5	128.47
**18**	80	0.5	10	8	78.47
**19**	20	0.5	10	2	159.60
**20**	5	0.5	40	2	110.32
**21**	80	0.5	2.5	2	55.75
**22**	20	0.5	40	0.5	158.27
**23**	20	0.5	2.5	8	43.45
**24**	5	0.5	10	8	43.45
**25**	20	0.5	10	2	138.73
**26**	20	0.5	2.5	0.5	142.67
**27**	5	0.125	10	2	65.13
**28**	80	0.5	40	2	85.54
**29**	80	0.5	10	0.5	145.08

Table S2. ANOVA for the quadratic RSM model (Box–Behnken). Model and terms related with sums of squares, F and p statistics (linear, interactions, quadratic), residuals, and lack-of-fit test (not significant at p=0.0705).

Source	Sum of Squares	df	Mean Square	F-Value	p-Value	
**Model**	46084.29	14	3291.74	14.07	< 0.0001	Significant
**ME**-Malt Extract	672.74	1	672.74	2.88	0.1120	
**G**-Glucose	267.12	1	267.12	1.14	0.3033	
**YE**-Yeast Extract	4748.58	1	4748.58	20.30	0.0005	
**DAP**-Diammonium phosphate	14937.97	1	14937.97	63.87	< 0.0001	
AB	146.31	1	146.31	0.6256	0.4422	
AC	239.63	1	239.63	1.02	0.3286	
AD	8.56	1	8.56	0.0366	0.8510	
BC	477.16	1	477.16	2.04	0.1751	
BD	1354.63	1	1354.63	5.79	0.0305	
CD	493.78	1	493.78	2.11	0.1683	
A^2	9145.07	1	9145.07	39.10	< 0.0001	
B^2	16203.01	1	16203.01	69.28	< 0.0001	
C^2	4717.77	1	4717.77	20.17	0.0005	
D^2	2199.93	1	2199.93	9.41	0.0084	
**Residual**	3274.39	14	233.89			
Lack of Fit	3025.49	10	302.55	4.86	0.0705	Not significant
Pure Error	248.90	4	62.23			
**Cor Total**	49358.68	28				

Table S3. Model summary statistics and lack-of-fit tests for sequential model building (linear, 2FI, quadratic, cubic). The quadratic model was selected based on R^2_{pred}.

Source	Sequential p-value	Lack of Fit p-value	R^2	Adjusted R^2	Predicted R^2	
Linear	0.0091	0.0039	0.4179	0.3209	0.2418	
2FI	0.9215	0.0025	0.4730	0.1802	-0.0496	
Quadratic	<0.0001	0.0705	0.9337	0.8673	0.6391	Suggested
Cubic	0.5624	0.0283	0.9700	0.8601	-2.5990	Aliased

Table S4. NMR spectroscopic data of Altenusin (^1H NMR in 700 MHz, ^{13}C NMR in 176 MHz, δ in ppm; DMSO-d_6)

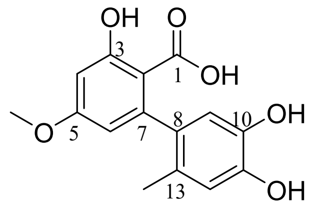

Position	δ_C	δ_H (J in Hz)	HMBC
1	171.87		
2	109.50		
3	161.89		
4	99.86	6.40, d (2.5)	C-2, C-5, C-6
5	162.11		
5-OCH_3	55.59	3.73, s	C-5
6	109.05	6.06, d (2.5)	C-4, C-7, C-8
7	145.19		
8	132.90		
9	116.13	6.41, s	C-7, C-10, C-11, C-13
10	144.11		
11	142.36		
12	116.95	6.51, s	C-8, C-10, C-11,13-CH_3
13	121.35		
13-CH_3	19.17	1.84, s	C-8, C-11, C-12, C-13

Table S5. Nonlinear regression parameters for Altenusin inhibition of mushroom tyrosinase using L-tyrosine and L-DOPA as substrates. Dose-response curves were fitted by nonlinear regression to a variable-slope sigmoidal (Hill-type) model, yielding the lower and upper plateaus of residual activity ("Bottom" and "Top", expressed as % of uninhibited control), IC_{50} values (μM), Hill slopes and coefficients of determination (R^2). n indicates the number of data points included in each fit.

**Substrate**	**n**	**Bottom (%)**	**Top (%)**	**IC_{50} (μM)**	**Hill slope**	**R^2**
**L-tyrosine**	12	-0.07 ± 0.50	100.57 ± 0.32	381.43 ± 2.44	-5.56 ± 0.16	0.9996
**L-DOPA**	12	-1.79 ± 1.29	123.41 ± 6.32	162.01 ± 23.22	0.93 ± 0.08	0.9935

Table S6. Michaelis–Menten kinetic parameters for mushroom tyrosinase in the absence and presence of Altenusin. Initial velocities for monophenolase (L-tyrosine) and diphenolase (L-DOPA) reactions were measured at varying substrate concentrations and fixed Altenusin concentrations ([I]) and fitted by nonlinear regression to the Michaelis–Menten equation. The table reports the apparent maximum velocity (V_{max}), Michaelis constant (K_m), their standard errors, and the coefficients of determination (R^2) for each fit. n denotes the number of substrate concentrations used in each regression.

Substrate	[I] (μM)	n	V_{max} (mM·s^{-1})	Error V_{max}	K_m (mM)	Error K_m	R^2
L-tyrosine	0	8	0.000668	0.000007	0.3864	0.0144	0.9964
	300	8	0.000668	0.000007	0.5634	0.0017	0.9955
	350	8	0.000668	0.000007	0.7068	0.0066	0.9849
	400	8	0.000668	0.000007	0.9315	0.0055	0.9938
L-DOPA	0	8	0.002365	0.000041	0.3549	0.0314	0.9828
	100	8	0.002011	0.000018	0.3866	0.0131	0.9850
	150	8	0.001873	0.000035	0.4400	0.0217	0.9787
	200	8	0.001731	0.000015	0.4787	0.0161	0.9929

Table S7. Lineweaver–Burk fits and competitive/mixed-inhibition parameters for Altenusin inhibition of mushroom tyrosinase (L-tyrosine and L-DOPA as substrate). Double-reciprocal (Lineweaver–Burk) plots were fitted by linear regression at different Altenusin concentrations ([I]), yielding the slope, y-intercept, apparent maximum velocity ($V_{max,app}$), apparent Michaelis constant ($K_{m,app}$) and coefficients of determination (R^2). Competitive and mixed-inhibition parameters (α, α′, K_i and K_i') were derived from the changes in $V_{max,app}$ and $K_{m,app}$ as a function of [I]; mean K_i and K_i' values are also reported.

Substrate	[I] (µM)	Slope	y-intercept	R^2	$V_{max,app}$ (mM·s-[1])	$K_{m,app}$ (mM)	Inhibition Type	α		K_i (µM)			
								α	α´	K_i	K_i´	Mean K_i	Mean K_i´
L-tyrosine	0	578.315985	1496.083492	0.997381	0.000668	0.386553	Competitive	-	-	-	-	470.9	-
	300	832.750928	1498.070212	0.996538	0.000668	0.555882		1.439958	-	681.882736	-		
	350	1048.470157	1513.136352	0.991817	0.000661	0.692912		1.812971	-	430.519618	-		
	400	1348.958073	1521.660185	0.995059	0.000657	0.886504		2.332562	-	300.173579	-		
L-DOPA	0	152.367980	422.081080	0.983337	0.002369	0.360992	Mix	-	-	-	-	253.4	606.2
	100	206.809393	484.581450	0.982205	0.002064	0.426779		1.357302	1.148077	279.875136	675.325727		
	150	246.319015	529.861924	0.974769	0.001887	0.464874		1.616606	1.255356	243.267111	587.415719		
	200	280.846011	573.933241	0.990774	0.001742	0.489336		1.843209	1.359770	237.189156	555.910535		

Table S8. Stern–Volmer and modified Stern–Volmer parameters for the interaction between Altenusin and mushroom tyrosinase. Stern-Volmer plots of fluorescence quenching at different temperatures (T) were fitted by linear regression to obtain the Stern-Volmer constants (K_{SV}) and coefficients of determination (R^2). Assuming a typical protein fluorescence lifetime ($\tau_0 = 10^{-8}$ s), bimolecular quenching constants (K_q) were calculated as K_{SV}/τ_0. Modified Stern-Volmer (double-log) analysis yielded apparent association constants (K_a) and binding site numbers (n) for Altenusin binding to tyrosinase.

T (K)	R^2 Stern-Volmer fit	K_{sv} (M^{-1})	Kq ($L·M^{-1}·s^{-1}$)	R^2 Double constant fit	K_a (M^{-1})	n
**298**	0.9947	$1.48×10^3$	$1.48×10^{11}$	0.9984	$1.64×10^3$	0.98
**303**	0.9898	$2.31×10^3$	$2.31×10^{11}$	0.9985	$2.56×10^3$	0.98
**310**	0.9954	$3.39×10^3$	$3.39×10^{11}$	0.9973	$4.44×10^3$	0.96

Table S9. Nonlinear regression parameters for Altenusin and reference compounds in copper-chelation, copper(II)-reduction and radical-scavenging assays. Dose–response curves for Altenusin, EDTA, L-ascorbic acid and Trolox were fitted by nonlinear regression to a variable-slope sigmoidal (Hill-type) model in the copper-chelation, copper(II)-reduction (BCS), DPPH and ABTS assays. The table lists the lower and upper plateaus of the fitted curves ("Bottom" and "Top", expressed as % of the uninhibited control), IC_{50} values (µM), Hill slopes and coefficients of determination (R^2). n denotes the number of experimental points included in each fit.

Assay	Compound	n	Top (%)	Bottom (%)	IC_{50} (µM)	Hill slope	R^2
**Copper - chelation**	Altenusin	12	110.90 ± 2.92	1.72 ± 1.05	905.63 ± 42.94	1.50 ± 0.11	0.9996
	EDTA	12	100.16 ± 0.62	0.82 ± 0.97	16.17 ± 0.27	3.68 ± 0.21	0.9935
**Copper (II) - Reduction**	Altenusin	12	104.01 ± 2.79	-2.05 ± 1.95	259.82 ± 15.91	1.97 ± 0.20	0.9956
	L-Ascorbic acid	12	104.22 ± 2.13	-0.25 ± 1.35	164.81 ± 10.94	1.45 ± 0.11	0.9945
**DPPH**	Altenusin	12	97.55 ± 3.42	5.24 ± 1.75	31.68 ± 2.04	2.59 ± 0.45	0.9947
	Trolox	12	97.87 ± 4.26	3.39 ± 1.45	36.16 ± 2.25	2.33 ± 0.84	0.9946
**ABTS**	Altenusin	11	101.58 ± 1.45	-0.01 ± 1.75	66.31 ± 1.53	1.79 ± 0.12	0.9994
	Trolox	11	98.03 ± 4.52	-0.71 ± 2.41	22.09 ± 0.13	2.00 ± 0.05	0.9976
